# Integrated meta-analysis and network pharmacology analysis: evaluation of Zhigancao decoction as treatment for diabetic cardiomyopathy

**DOI:** 10.3389/fcvm.2025.1454647

**Published:** 2025-03-14

**Authors:** Kangshou Ji, Meizi Han, Mingqian Yang, Qian Xu, Yan Zhang

**Affiliations:** ^1^ First Clinical College, Liaoning University of Traditional Chinese Medicine, Shenyang, China; ^2^ Department of Cardiovascular Medicine, Affiliated Hospital of Liaoning University of Traditional Chinese Medicine, Shenyang, China; ^3^ National Key Laboratory of Chinese Medicine Modernization, Heilongjiang University of Traditional Chinese Medicine, Harbin, China; ^4^ Chinese Medicine College, Liaoning University of Traditional Chinese Medicine, Shenyang, China

**Keywords:** diabetic cardiomyopathy, Zhigancao decoction, meta-analysis, network pharmacology, AGEs/RAGE signaling pathway

## Abstract

**Background:**

Zhigancao Decoction (ZGCD) is derived from “Treatise on Febrile Diseases” and is traditionally prescribed for treating a variety of cardiovascular conditions. As of now, there are no data to support its use as a treatment for diabetic cardiomyopathy (DCM) and the mechanism behind the effect is unclear as well. In the present study, clinical evidence for the efficacy of ZGCD in patients with DCM was examined using a meta-analysis and its underlying anti-DCM molecular mechanisms were explored via network pharmacology.

**Methods:**

The current study utilized an extensive search strategy encompassing various domestic and foreign databases databases to retrieve pertinent articles published up to June 2024. In light of this, a thorough evaluation of the benefits and safety of Zhigancao decoction (ZGCD) was conducted in this study using RevMan and Stata. Subsequently, a number of active compounds and target genes for ZGCD were gathered from the TCMSP and BATMAN-TCM databases, while the main targets for DCM were obtained from databases such as GenCards, OMIM, TTD, and DrugBank. To select core genes, protein-protein interaction networks were generated using the STRING platform, and enrichment analyses were completed using the Metascape platform.

**Results:**

Meta-analysis results were ultimately derived from 9 studies involving 661 patients in total. In comparison with WM therapy alone, the pooled results showed that ZGCD significantly enhanced overall effectiveness. Additionally, the utilization of ZGCD was leading to a reduction in LVEDV, LVESV and LVDD, also a greater increase in LVEF. Meanwhile, the utilization of ZGCD during intervention was more effective in reducing SBP, and DBP. In addition, the ZGCD showed potential in reducing the occurrence of adverse events. In the context of network pharmacology, five constituents of ZGCD—namely lysine, quercetin, gamma-aminobutyric acid, stigmasterol, and beta-sitosterol—are posited to exert anti-diabetic cardiomyopathy (anti-DCM) effects through interactions with the molecular targets ASS1, SERPINE1, CACNA2D1, AVP, APOB, ICAM1, EGFR, TNNC1, F2, F10, IGF1, TNNI2, CAV1, INSR, and INS. The primary mechanisms by which ZGCD may achieve its anti-DCM effects are likely mediated via the AGEs/RAGE signaling pathway, as well as through pathways related to lipid metabolism and atherosclerosis.

**Conclusion:**

In comparison to WM therapy alone, ZGCD demonstrates greater efficacy and safety in the management of DCM. ZGCD not only significantly reduces blood pressure, but also enhances cardiac function while producing fewer adverse effects. The therapeutic effects of ZGCD on DCM can likely be ascribed to its capacity to modulate the AGEs-RAGE signaling pathway, as well as its efficacy in enhancing lipid metabolism and mitigating atherosclerosis.

**Systematic Review Registration:**

identifier (INPLASY202430133).

## 1 Introduction

Diabetes mellitus (DM) is a complex endocrine disorder marked by elevated blood glucose levels and disruptions in carbohydrate, lipid, and protein metabolism. The incidence of diabetes has skyrocketed across the globe and has become the largest proportion of the disease burden of the elderly in China (1). It is projected that approximately 439 million individuals will be affected by type 2 diabetes mellitus (T2DM) by the year 2030, posing substantial economic challenges for individuals, healthcare systems, and nations (2). A variety of organs, such as the heart, brain, liver, and kidneys,may become dysfunctional as a result of this condition, ultimately resulting in unfavorable health outcomes (3).Cardiovascular issues are the primary source of illness and death in diabetic individuals (4). Patients with T2DM face a significantly elevated risk, ranging from 2 to 4 times higher than non-diabetic individuals, of developing cardiovascular disease due to diabetes-related coronary atherosclerosis and vascular abnormalities (5, 6). Thus, the prevention or postponement of cardiovascular complications in diabetic patients is imperative.

Diabetic cardiomyopathy (DCM) was first proposed in 1972 by Rubler and is a disorder of the heart muscle in patients with diabetes (7, 8). In diabetes mellitus patients, prolonged hyperglycemia may lead to a set of functional and structural changes, initially presenting with diastolic relaxation abnormalities and progressing to systolic dysfunction, characterized by cardiomyocyte stiffness, fibrotic alterations, and cardiomyocyte apoptosis, ultimately leading to substantial myocardial necrosis, culminating in heart failure and potentially cardiogenic shock (9, 10). Epidemiological data indicates a prevalence of 16.9% of diabetic cardiomyopathy among individuals with diabetes, with corresponding mortality and disability rates of approximately 18% and 22%, respectively (11, 12). Basically, DCM is one of the end-stage consequences of mortality and morbidity in patients with DM. In recent years, the primary research focus of DCM is how to delay and prevent its occurrence and development, and now established that the pathogenesis of DCM is complex and multifactorial, including oxidative stress (13, 14), autophagy (15), myocardial apoptosis (16) and so on.

Currently, there is no specific and effective treatment available for DCM. The prevailing clinical management primarily depends on glucose-lowering therapies and symptomatic supportive care. Previously reported large randomized controlled studies, comprising the United Kingdom Prospective Diabetes Study (UKPDS) (17), the Action to Control Cardiovascular Risk in Diabetes (ACCORD) study (18), Action in Diabetes and Vascular Disease (ADVANCE) (19) and the Veterans Affairs Diabetes Trial (VADT) (20), showed that it is imperative to adhere to a stringent blood glucose control regimen in order to mitigate the development and advancement of cardiovascular diseases, myocardial infarction, and mortality. While recent advancements in glucose-lowering medications have facilitated optimal blood glucose management in individuals with diabetes, emerging studies indicate that glycemic control alone may not sufficiently mitigate cardiovascular complications during the intermediate and advanced stages of T2DM (21). Grievously, the current knowledge regarding the pathogenesis of DCM remains limited, leading to a lack of effective and targeted therapeutic options. The prevailing approach to managing DCM primarily centers on addressing multiple risk factors, encompassing blood glucose regulation and cardiovascular improvement. The complexity of multi-drug regimens may increase the risk of patient non-compliance, and long-term use of hypoglycemic agents can result in various adverse effects such as weight gain, gastrointestinal issues, and hepatic dysfunction. Due to the less than optimal results associated with current treatment modalities, healthcare professionals are increasingly exploring traditional Chinese medicine (TCM) as an adjunctive therapeutic option.

With a history spanning over two thousand years, TCM is regarded as a significant component of Chinese cultural heritage. Fundamentally, TCM emphasizes the attainment of balance between individuals and their surroundings, while advocating for a holistic treatment approach. The patient-focused, holistic, and multi-faceted strategies utilized in TCM have demonstrated unique advantages in addressing complex conditions such as diabetes mellitus (22). In TCM literature, there is no specific disease name for DCM, but based on its clinical manifestations and features, it is categorized as “Xiao Xin”. Zhigancao Decoction (ZGCD), also known as Fumai Decoction, a TCM formulation known for its ability in reinforcing Qi, supplementing blood, and regulating pulse, originates from the “Treatise on Febrile Diseases” written during the Han Dynasty by Zhang Zhongjing. In recent years, there has been an increasing focus on the therapeutic efficacy of Zhigancao decoction, which has been extensively utilized in the management of arrhythmia (23), chronic heart failure (24), myocardial fibrosis, and atrial fibrillation (25). Meanwhile, researchers have further investigated its impact on cardiac electrophysiology (26). Regarding the treatment of DCM, despite the growing body of preclinical and clinical evidence supporting the efficacy of ZGCD, the majority of published clinical trials are constrained in scope, consisting of small-scale single-center studies that lack thorough systematic assessment and review of clinical interventions. Consequently, evidence-based research is necessary to confirm the efficacy and safety profile of ZGCD.

Building on the aforementioned background, ZGCD exhibits a broad spectrum of anti-DCM efficacy, yet lacks sufficient clinical evidence, and its mechanism remains elusive due to the intricate nature of traditional Chinese medicine compound ingredients. Therefore, the aim of this research is to assess the clinical data on the efficacy of ZGCD in DCM patients through a meta-analysis, and to explore its potential anti-DCM molecular mechanisms using network pharmacology.

## 2 Materials and methods

### 2.1 Meta-analysis

The review procedure was carried out in accordance with PRISMA guidelines, and it has been submitted to the International Platform of Registered Systematic Review and Meta-analysis Protocols (INPLASY) under registration number INPLASY202430133.

#### 2.1.1 Outline of literature search

A comprehensive database search was conducted to assess herbal therapeutic interventions for DCM, such as PubMed, EMBASE, Web of Science, and Cochrane Library, as well as China National Knowledge Infrastructure (CNKI), Wanfang Database, Chinese Scientific Journals Database (VIP) and Chinese Biomedical Literature Database (CBM). Within the study scope, data retrieval covered the entire duration of all databases up until March 2024, without regard to language, participant condition, or publication year. Keywords and MeSH terms were combined in the present search strategy, with particular focus on “Diabetic cardiomyopathy” and “Zhigancao Decoction”. Moreover, the search encompassed interventions and diseases pertinent to the subjects under investigation, such as Zhigancao Soup, Fumai Decoction and DCM etc. Furthermore, a manual search of journal literature was conducted to complement the initial search and address any potential oversights. The comprehensive search strategy for each database can be found in Supplementary Material S1.

#### 2.1.2 Inclusion criteria

(1) Types of Participants. Neither age, gender nor race was considered in the study. Individuals with DCM diagnosed on the basis of internationally recognized diagnostic criteria or a well-defined definition participated in the study.
(2) Types of Interventions. The present study exclusively implemented ZGCD as the positive intervention in the ZGCD group, as opposed to the control group. Dosages and durations of treatment were not restricted.
(3) Types of Comparison. The efficacy of Western medicine (WM) treatment in reducing blood glucose levels and improving cardiovascular function has been demonstrated. In these studies, there were no differences in the specifications and dosage of WM between the control and ZGCD groups.
(4) Types of Outcomes. The primary endpoints were the total effective rate and adverse reactions, with secondary outcomes including the left ventricular volumes at end diastole (LVEDV) and end systole (LVESV), left ventricular ejection fraction (LVEF), left ventricular diastolic diameter (LVDD), systolic (SBP) and diastolic blood pressure (DBP) and so on. A minimum of one result has been reported from every article included in the review.
(5) Types of Study Design. This study included all controlled trials that reported the use of ZGCD for the treatment of DCM, regardless of their publication status or language.

#### 2.1.3 Exclusion criteria

The exclusion criteria were established as follows: (1) Studies that were not clinical trials or involved animal subjects. (2) Studies in which the control group utilized traditional Chinese medicine modalities, including Chinese patent medicine, acupuncture, herbal extracts, and similar interventions. (3) Studies that presented duplicated publications or redundant clinical data. (4) Studies containing original or data could not be accessed or extracted despite attempts to contact the authors. (5) Studies in which the outcome effect was unclear due to incomplete data, lack of clarity in the reported outcomes, incorrect statistical methods, etc.

#### 2.1.4 Baseline characteristics of studies

A team of two independent individuals extracted the data for the studies. In order to enhance efficiency, different variables such as first authors, dates of publication, countries, study designs, sample sizes, average ages, genders, intervention measures, and duration of follow-up were organized in a study-specific Excel spreadsheet. Subsequently, the data underwent cross-validation and were imported into Review Manager. The risk of bias in each study included in the analysis was evaluated using the Cochrane Handbook for Systematic Reviews. In instances of disagreement, a third reviewer was consulted to achieve consensus.

#### 2.1.5 Statistical analysis

All analyses were conducted using Review Manager (version 5.4) along with Stata software (version 17.0). The odds ratio (OR) was used for binary variables, while the mean difference (MD) or standardized mean difference (SMD) was employed for continuous variables, depending on the units of measurement. 95% confidence intervals (CIs) were provided for the results. An evaluation of heterogeneity was conducted by using chi-square statistics, with a determination of its presence based on a *P*-value exceeding 0.1 or an *I*^2^ statistic below 50%, leading to the selection of the fixed-effects model. Conversely, a *P*-value equal to or less than 0.1 or an *I*^2^ statistic equal to or greater than 50% resulted in the utilization of the random effects model. As well, we conducted a sensitivity analysis to examine the stability of each outcome. For the purpose of determining publication bias, Begg's, Egger's and funnel plots were performed. Trial sequential analysis (TSA) was employed to determine the sufficiency of sample sizes for assessing the outcomes of the meta-analysis. The *p*-value of less than 0.05 was used to determine statistical significance.

#### 2.1.6 Certainty assessment of evidence

The assessment of outcome evidence quality was conducted utilizing the GRADE system, taking into account various factors including risk of bias, inconsistency, indirectness, imprecision, and publication bias. In order to rank the evidence quality, four levels were used: high, moderate, low, and very low.

### 2.2 Network pharmacology

#### 2.2.1 Screening for compounds and corresponding targets of ZGCD

The Traditional Chinese Medicine Systems Pharmacology Database and Analysis Platform (TCMSP) was utilized to identify the primary chemical components of the natural medicines in ZGCD. Subsequently, all screened ingredients were imported into the TCMSP for normalization according to ADME (absorption, distribution, metabolism, excretion) criteria. In addition, oral bioavailability ≥30% and drug-likeness ≥0.18 as the most commonly used pharmacokinetic parameters to measure drug properties. In cases where certain drugs were not found in TCMSP, the BATMAN-TCM database was employed for further investigation.The criteria for screening targets of BATMAN-TCM included a score cut-off of ≥20% and an adjusted *p*-value of 0.05 (*p*-value post Benjamini–Hochberg correction for multiple testing). Subsequent to this, genetic data pertaining to these targets was sourced from the Uniprot database, and human genetic information concerning these targets was retrieved for gene annotations.

#### 2.2.2 Target prediction for ZGCD associated with DCM

High correlation targets associated with DCM were identified by querying the related keywords in various public databases, including DisGNET, DrugBank, GeneCards, OMIM, and TTD. In order to predict ZGCD's therapeutic targets for the treatment of DCM, a comprehensive analysis was conducted to identify common targets between compounds and DCM. Specifically, and a Venn diagram was obtained using the Venn 2.1.0 platform. The intersection targets were entered into Cytoscape software (version 3.8.2) to construct Herb-Compound-Target (H-C-T) networks.

#### 2.2.3 Network construction of protein-protein interactions

Additionally, as a means of improving understanding of core regulatory targets in treating DCM and to identify potential relationships, the intersection targets were input into the STRING database (Version: 12.0) to construct a protein-protein interaction (PPI) network. The screening criteria included selecting human as the species, a score greater than 0.9, and hidden independent protein molecules. The above results were identified as core intersection targets and imported into Cytoscape software (version 3.8.2). Furthermore, use of the CytoNCA plugin enabled us to determine the “betweenness centrality” (BC), the “closeness centrality” (CC), and the “degree” (DC) of each node. Hub genes with values exceeding the mean were identified through computations.

#### 2.2.4 Go and KEGG enrichment analysis

A KEGG and GO enrichment assessment based on the core intersection targets identified in Section 2.2.3 is later conducted on the Metascape website. In both the GO enrichment analysis and the KEGG pathway analysis, the top 10 records showing a *p* value < 0.05 were chosen for visualization.

## 3 Results of meta-analysis

### 3.1 Overview of literature retrieval

**Figure 1:**
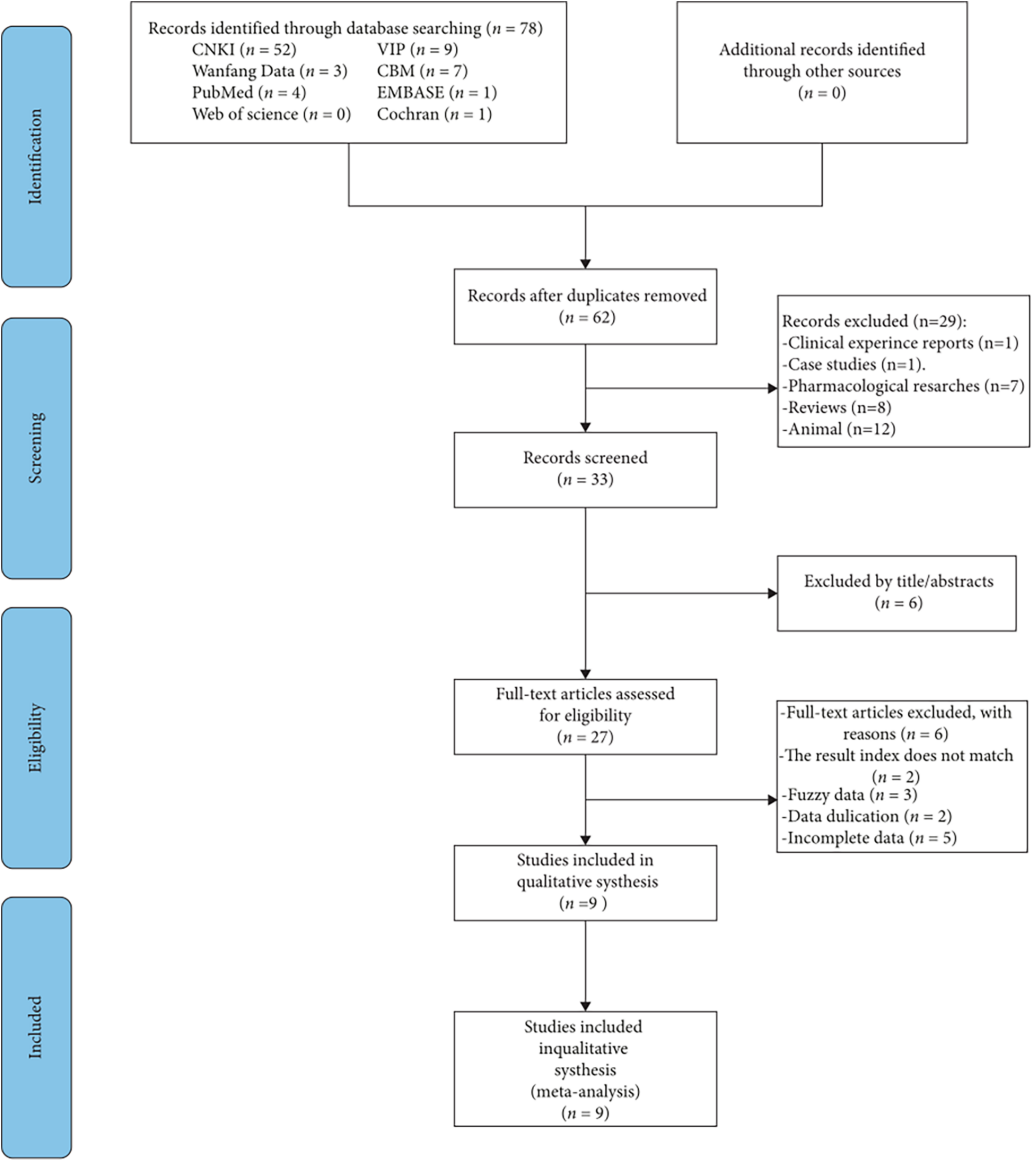
Overview of the study selection process.

A total of 81 articles were initially reviewed, with 9 articles (27–35) meeting the inclusion criteria. The search procedure is detailed in Figure 1.

### 3.2 Description of study characteristics and bias risk

**Figure 2:**
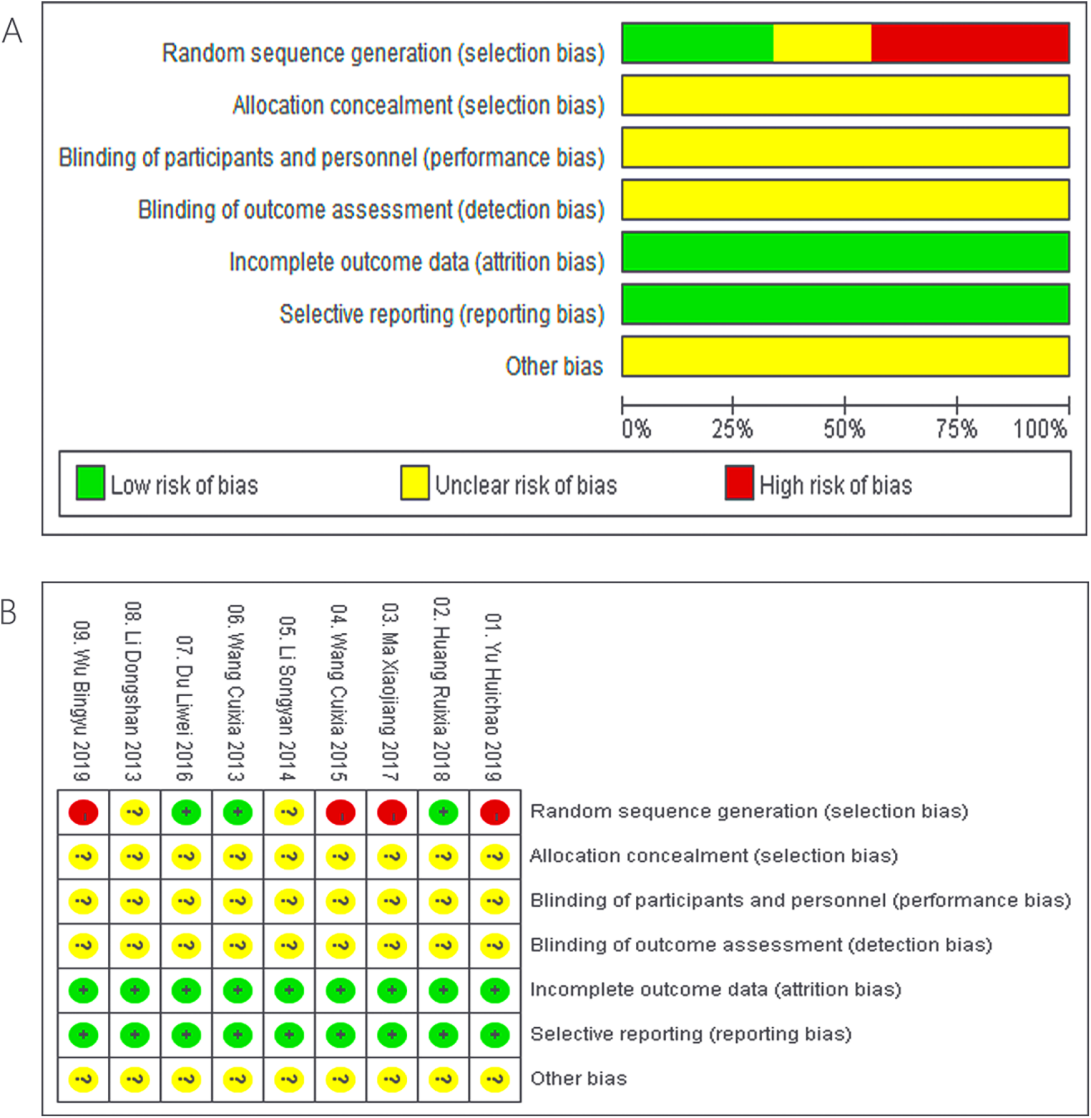
Risk of bias graph **(A)**, and risk of bias summary **(B)**.

The present meta-analysis incorporated 9 studies comprising a total of 661 individuals, with 333 cases in the ZGCD group and 328 cases in the control group (Table 1 and Supplementary Material S2). The risk of bias was assessed using Rev Man 5.4 software (Figure 2).

**Table 1:** Summary of included studies.

No.	Author (year)	Sample size E/C	Gender(M/F)		Mean age (y)		Intervention		Duration (weeks)	Outcomes
			E	C	E	C	E	C		
1	Yu Huichao 2019 (27)	53/51	33/20	29/22	59.12 + 2.33	58.44 + 2.26	ZGCD + C	CWM + ST	NA	①③
2	Huang Ruixia 2018 (28)	39/39	14/25	15/24	62.84 + 10.92	62.72 + 11.06	ZGCD + C	CWM + ST	2	①③
3	Ma Xiaojiang 2017 (29)	30/30	11/19	15/15	65.57 + 4.82	65.98 + 4.24	ZGCD + C	CWM + ST	2	①④
4	Wang Cuixia 2015 (30)	16/14	4/12	5/9	64 + 12	62 + 13	ZGCD + C	CWM	2	①②
5	Li Songyan 2014 (31)	40/40	27/53		63.4		ZGCD + C	CWM + ST	NA	①
6	Wang Cuixia 2013 (32)	30/30	10/20	10/20	60 + 10	60 + 18	ZGCD + C	CWM + ST	2	①④
7	Du Liwei 2016 (33)	47/46	24/23	23/23	58.9 + 3.1	59 + 2.8	ZGCD + C	CWM + ST	NA	①③
8	Li Dongshan 2013 (34)	38/38	46/30		62 + 1.3		ZGCD + C	CWM + ST	2	①③④
9	Wu Bingyu 2019 (35)	40/40	15/25	16/24	62.7 + 1.2	62.5 + 1.1	ZGCD + C	CWM	2	①②④

ZGCD, Zhigancao decoction; CWM, common western medicine; ST, specific therapy.

Outcomes: ① Total effective rate; ② Cardiac function indicators: LVEDV, LVESV, LVEF, LVDD; ③ AD indicators: AD rate, and hypotension. ④ BP:SBP, and DBP.

### 3.3 Meta-analysis results

#### 3.3.1 Total effective rate

**Figure 3:**
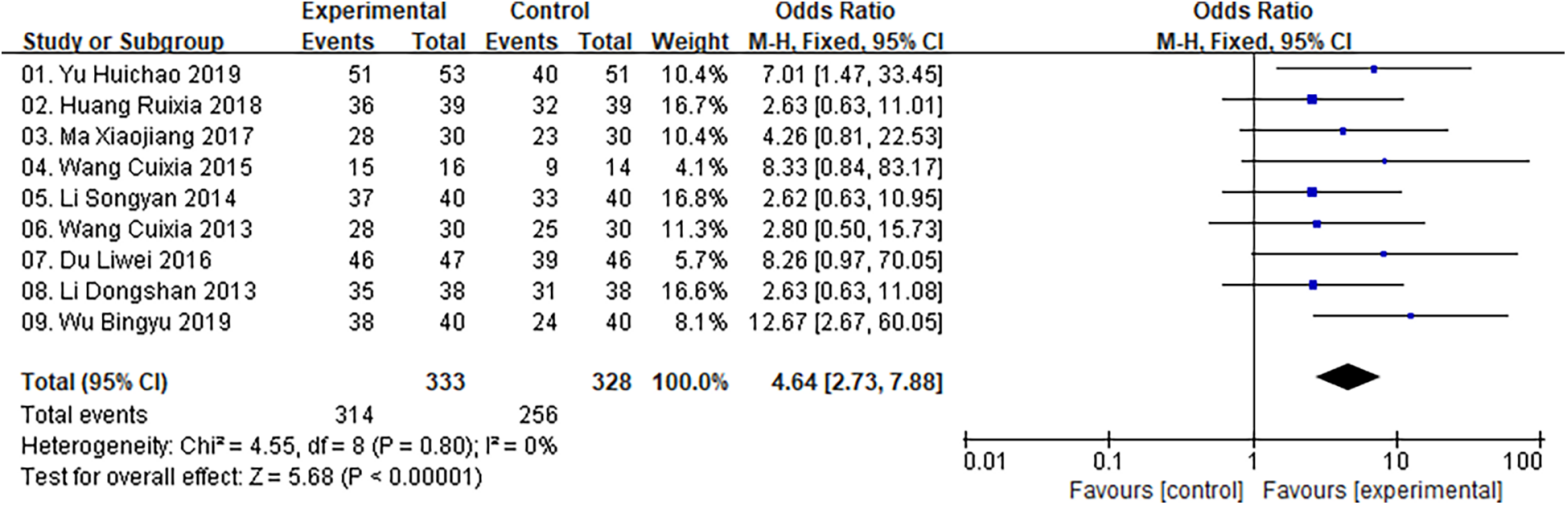
Meta result of total effective rate.

The present analysis covered 9 studies with 661 patients in total. The result of heterogeneity test showed that there was no heterogeneity across studies (Chi^2^ = 4.55, *P* = 0.80, *I*^2^ = 0%), and thus a fixed-effects model was utilized for statistics. The findings indicated that the ZGCD group exhibited a statistically significant enhancement in overall effective rate when contrasted with the control groups [OR = 4.64, 95% CI (2.73, 7.88), *P* < 0.00001] (Figure 3).

#### 3.3.2 Cardiac function indicator

**Figure 4:**
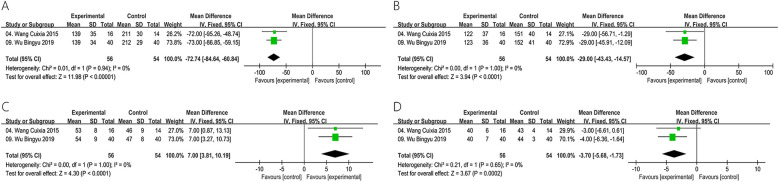
Meta result of LVEDV **(A)**, LVESV **(B)**, LVEF **(C)**, LVDD **(D)**.

Two studies encompassing a total of 110 patients evaluated LVEDV, LVESV, LVEF and LVDD. The result of heterogeneity test showed that there was no heterogeneity regarding LVEDV (Chi^2^ = 0.01, *P* = 0.94, *I*^2^ = 0%), LVESV (Chi^2^ = 0.00, *P* = 1.00, *I*^2^ = 0%), LVEF (Chi^2^ = 0.00, *P* = 1.00, *I*^2^ = 0%) and LVDD (Chi^2^ = 0.21, *P* = 0.65, *I*^2^ = 0%), and thus the fixed effects model was chosen. The outcomes revealed that, the utilization of ZGCD during intervention was related to a reduction in LVEDV [SMD = −72.74, 95% CI (−84.64, −60.84); *P* < 0.00001] (Figure 4A), a reduction in LVESV [SMD = −29.00, 95% CI (−43.43, −14.57); *P* < 0.00001] (Figure 4B), a reduction in LVDD [SMD = −3.70, 95% CI (−5.68, −1.73); *P* = 0.0002] (Figure 4C), as well as a a greater increase in LVEF [SMD = 7.00, 95% CI (3.81, 10.19); *P* < 0.00001] (Figure 4D).

#### 3.3.3 Blood pressure

**Figure 5:**
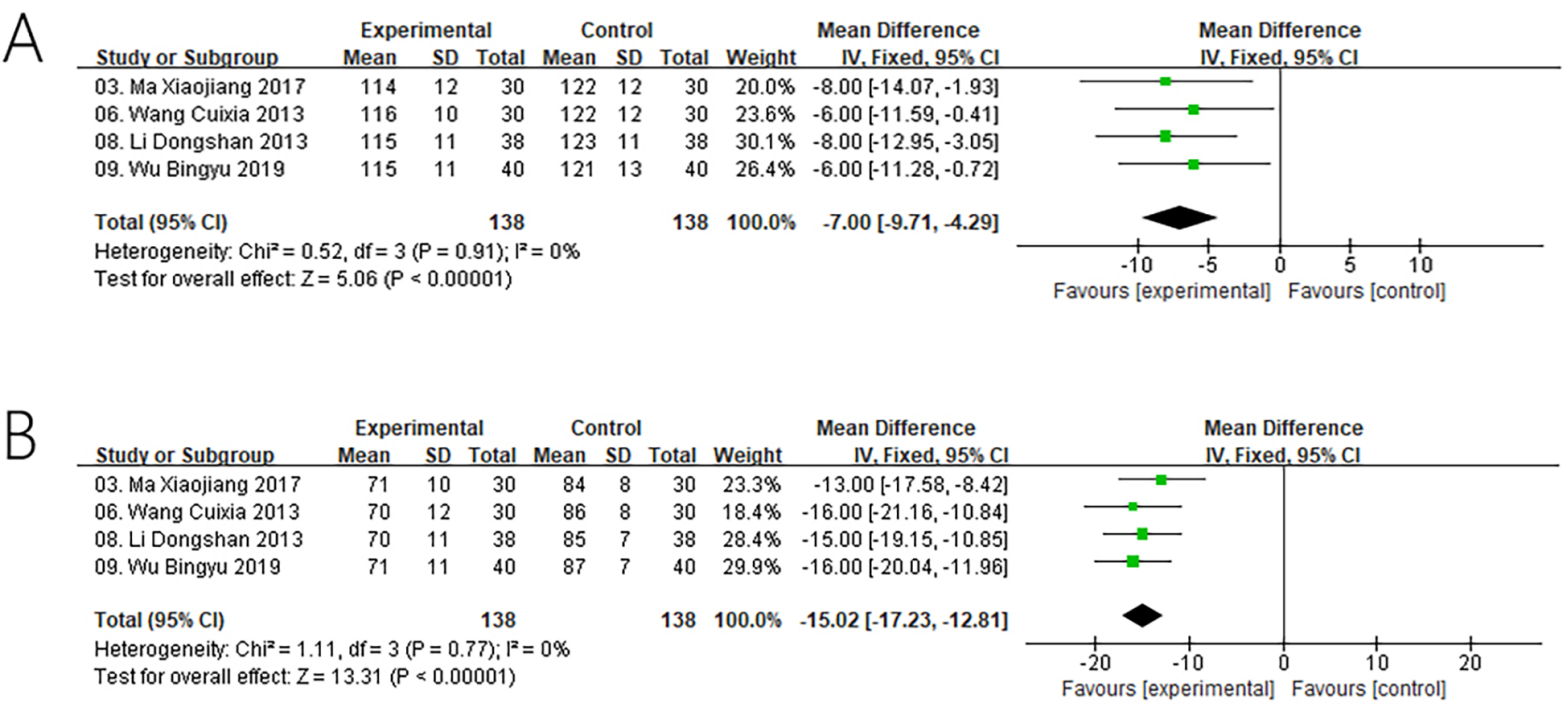
Meta result of systolic blood pressure **(A)** and diastolic blood pressure **(B)**.

A total of 4 studies evaluated systolic blood pressure (Figure 5A), and 4 studies evaluated diastolic blood pressure (Figure 5B). The result of heterogeneity test showed that there was no heterogeneity across studies regarding systolic blood pressure (Chi^2^ = 0.52, *P* = 0.9, *I*^2^ = 0%)and diastolic blood pressure (Chi^2^ = 1.11, *P* = 0.77, *I*^2^ = 0%), so the fixed-effects model was employed for analysis. The outcomes revealed that, in comparison to the control groups, the utilization of ZGCD during intervention was more effective in reducing systolic blood pressure [SMD = −7.00, 95% CI (−9.71,−4.29); *P* < 0.00001], and diastolic blood pressure [SMD = −15.02, 95% CI (−17.23 −12.81); *P* < 0.00001].

#### 3.3.4 Adverse reactions

**Figure 6:**
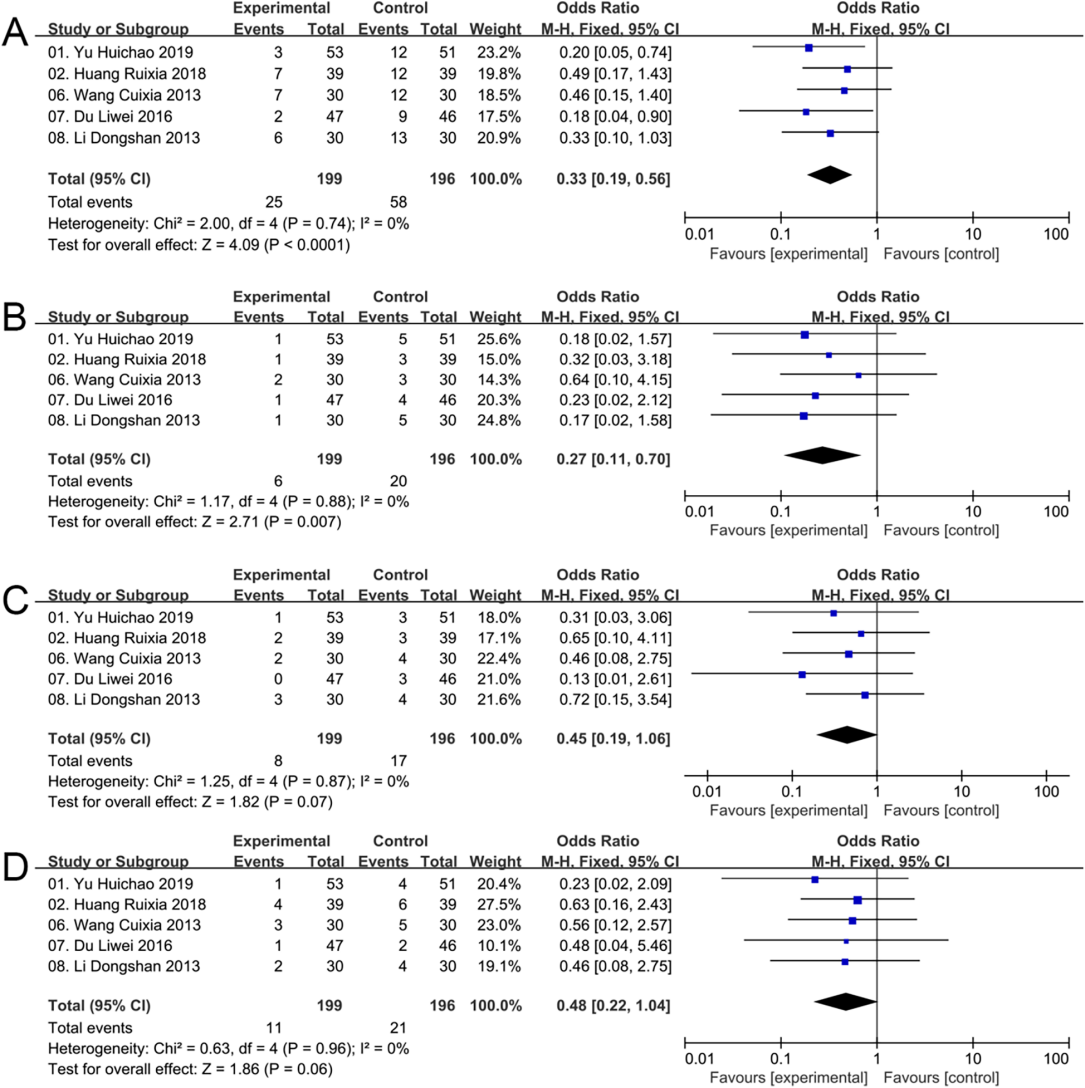
Meta result of adverse reactions **(A)**, hypotension **(B)**, headaches **(C)** and dizziness **(D)**.

Data on adverse events were collected from 5 studies. The result of heterogeneity test showed that there was no heterogeneity across studies (Chi^2^ = 1.17, *P* = 0.74, *I*^2^ = 0%), and thus a fixed-effects model was utilized for statistics. In the results, it was found that adverse events were significantly lower in the ZGCD groups than in the control groups [OR = 0.33, 95% CI (0.19, 0.56); *P* < 0.0001] (Figure 6A).

Specifically, three distinct adverse reactions were documented in the analysis of five studies. A total of 25 patients in the experimental group had adverse reactions during treatment, including 6 cases of hypotension, 8 cases of headache, and 11 cases of dizziness. In parallel, a total of 58 patients in the control group experienced adverse reactions, including 20 cases of hypotension, 17 cases of headache, and 21 cases of dizziness.

The result of heterogeneity test showed that there was no heterogeneity across studies regarding hypotension (Chi^2^ = 1.17, *P* = 0.88, *I*^2^ = 0%), headaches (Chi^2^ = 1.25, *P* = 0.87, *I*^2^ = 0%) and dizziness (Chi^2^ = 0.63, *P* = 0.96, *I*^2^ = 0%), so the fixed-effects model was employed for analysis. Statistical examination of hypotension was carried out, revealing a notably lower overall occurrence rate in the ZGCD groups compared to the control groups [OR = 0.27, 95% CI (0.11, 0.70); *P* = 0.007] (Figure 6B). Furthermore, the meta-analysis data suggested that the use of ZGCD was associated with a reduced incidence of headaches [OR = 0.45, 95% CI (0.19, 1.06); *P* = 0.07] (Figure 6C) and dizziness [OR = 0.48, 95% CI (0.22, 1.04); *P* = 0.06] (Figure 6D), however, these conclusions require further verification.

#### 3.3.5 Sensitivity analysis

**Figure 7:**
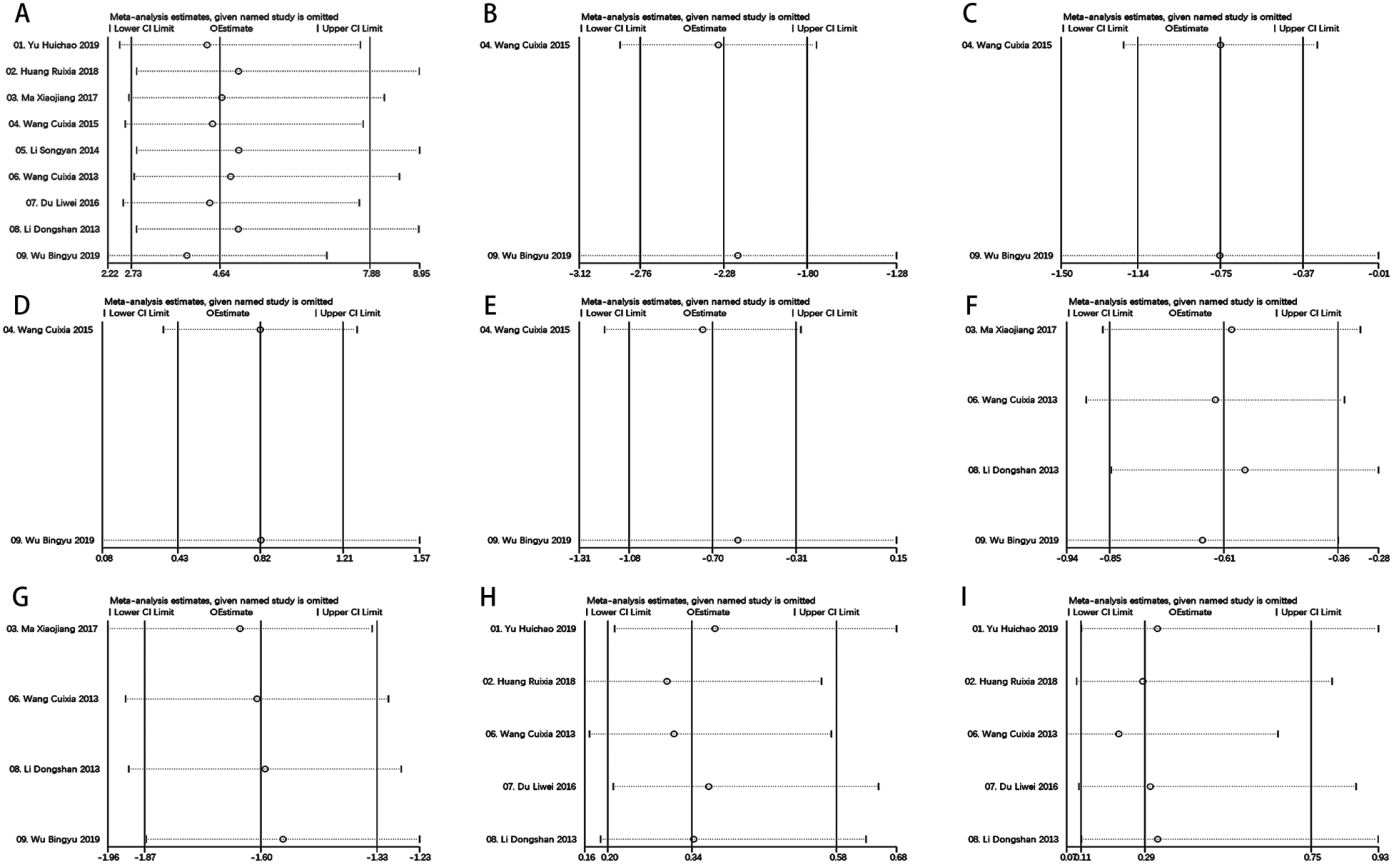
Metaninf results of each outcomes. **(A)** Total Effective Rate, **(B)** LVEDV, **(C)** LVESV, **(D)** LVEF, **(E)** LVDD, **(F)** SBP, **(G)** DBP, **(H)** Adverse Reactions **(I)** Hypotension.

To validate the strength and dependability of the analytical outcomes, a sensitivity analysis was executed through metaninf algorithm in Stata V14.0. Noteworthy alterations in the outcomes of the remaining trials were not detected upon the exclusion of each study, affirming the stability and credibility of the findings (Figure 7 and Supplementary Material S3).

#### 3.3.6 Publication bias

**Figure 8:**
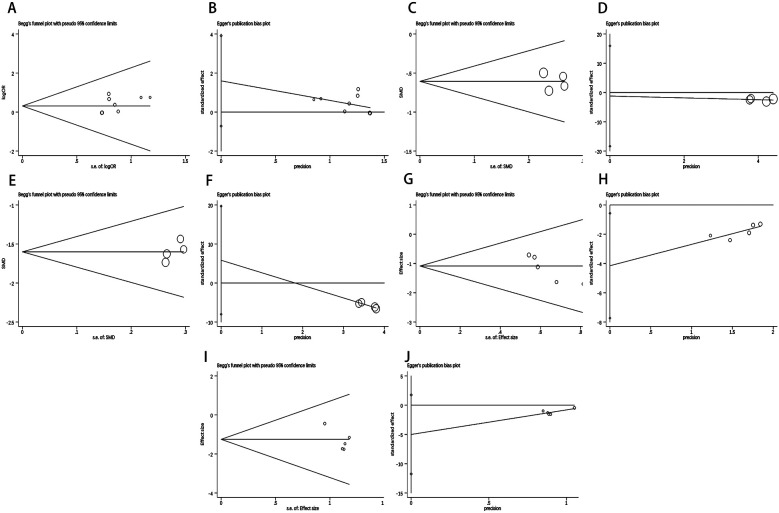
Results of Begg's test and Egger's test. Begg's test result of **(A)** Total Effective Rate, **(C)** SBP, **(E)** DBP, **(G)** Adverse Reactions, **(I)** Hypotension. Egger's test result of **(B)** Total Effective Rate, **(D)** SBP, **(F)** DBP, **(H)** Adverse Reactions, **(J)** Hypotension.

When more than 4 studies were included in the outcome indicators, Begg's and Egger's tests were used to determine publication bias, both yielding the *p*-value < 0.05 indicating publication bias. Figure 8 and Supplementary Material S3 illustrated that Begg's and Egger's tests show no publication bias for total effective rate, DBP, SBP, adverse reactions, and hypotension.

**Figure 9:**
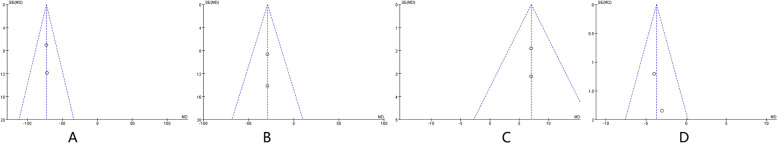
Funnel diagram of LVEDV **(A)**, LVESV **(B)**, LVEF **(C)** and LVDD **(D)**.

For LVEDV, LVESV, LVEF and LVDD, the funnel plots were used to assess their publication bias. Based on the results, the funnel showed simple symmetry on both sides, indicating no significant publication bias (Figure 9).

#### 3.3.7 Quality of evidence

Using GRADE as a method of evaluating evidence, the quality of evidence was evaluated. The result for each outcome ranged from moderate to poor, owing to the likelihood of bias and imprecision of findings. Detailed information about each outcome was presented in Table 2.

**Table 2:** GRADE evidence quality of outcomes included in the literature.

Quality assessment							No. of patients		Effect		Quality	Importance
No. of studies	Design	Risk of bias	Inconsistency	Indirectness	Imprecision	Other considerations	Experimental	Control	Relative	Absolute		
Total effective rate												
9	RCT	Serious^a^	No serious inconsistency	No serious indirectness	No serious imprecision	None	314/333 (94.3%)	256/328 (78%)	OR 4.64 (2.73–7.88)	162 more 1,000 (from 126 more to 185 more)	Moderate	Critical
								81.6%		138 more per 1,000 (from 108 more to 156 more)		
LVEDV												
2	RCT	Serious^a^	No serious inconsistency	No serious indirectness	Serious^b^	None	56	54	–	MD 72.74 lower (84.64 to 60.84 lower)	Low	Important
LVESV												
2	RCT	Serious^a^	No serious inconsistency	No serious indirectness	Serious^b^	None	56	54	–	MD 29.00 lower (43.43 to 14.57 lower)	Low	Important
LVEF												
2	RCT	Serious^a^	No serious inconsistency	No serious indirectness	Serious^b^	None	56	54	–	MD 7.00 higher (3.81 to 10.19 higher)	Low	Important
LVDD												
2	RCT	Serious^a^	No serious inconsistency	No serious indirectness	Serious^b^	None	56	54	–	MD −3.70 lower (−5.68 to 1.73 lower)	Low	Important
SBP												
4	RCT	Serious^a^	No serious inconsistency	No serious indirectness	No serious imprecision	None	138	138	–	MD 7.00 lower (9.71 to 4.29 lower)	Moderate	Important
DBP												
4	RCT	Serious^a^	No serious inconsistency	No serious indirectness	No serious imprecision	None	138	138	–	MD 15.02 lower (17.23 to 12.81 lower)	Moderate	Important
Adverse reaction												
4	RCT	Serious^a^	Serious^b^	No serious indirectness	Serious^c^	None	18/169 (10.7%)	46/166 (27.7%)	OR 0.30 (0.16–0.55)	174 fewer 1,000 (from 103 fewer to 219 fewer)	Moderate	Critical
								27.2%		171 more per 1,000 (from 102 fewer to 216 fewer)		
Hypotension												
4	RCT	Serious^a^	Serious^b^	No serious indirectness	Serious^c^	None	4/169 (2.4%)	17/166 (10.2%)	OR 0.21 (0.07 to 0.64)	79 fewer 1,000 (from 34 fewer to 94 fewer)	Moderate	Important
								9.3%		72 more per 1,000 (from 31 fewer to 86 more)		

^a^ The report lacks explanations of certain techniques for randomization, concealing allocations, and blinding.

^b^ Fewer included articles and observers.

^c^ The 95% Cl crosses the invalid line.

#### 3.3.8 Trial sequential analysis

The potential for false positive and false negative results in systematic reviews and meta-analyses can be mitigated through the use of Trial Sequential Analysis (TSA), a statistical method that has gained popularity. In this study, TSA 0.9.5.10 beta software was utilized to ensure the reliability and conclusiveness of the meta-analytic results. The required sample size for the research was calculated utilizing a significance level (*α*) of 0.05 (two-tailed) and a power of 80% (*β* = 0.20), as determined by the O'Brien-Fleming alpha-spending function. Using the mean event proportions for both experimental and control groups, the relative risk reductions and rates of events were calculated.

**Figure 10:**
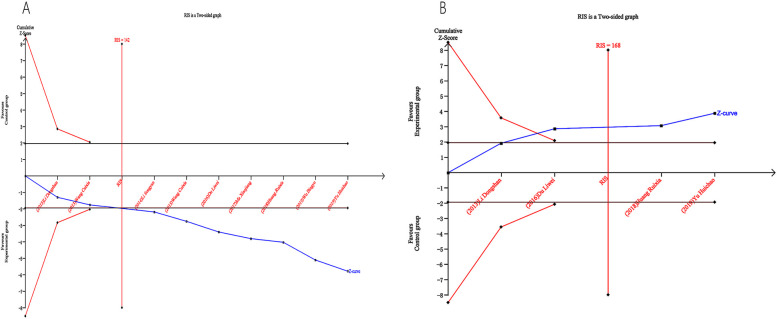
Trial sequential analysis (TSA) for total effective rate **(A)** and adverse reactions **(B)**.

The TSA analyzed 9 trials and found a controlled-event proportion of 78.04% and an RR reduction of −20.82% in terms of the total effective rate. Based on the TSA, the required information size (RIS) was determined as 142. The cumulative Z-curve surpassed the adjusted boundary of the RIS, indicating that ZGCD therapy would yield definitive and strong outcomes (Figure 10A).

The TSA analyzed 4 trials and found a controlled-event proportion of 27.71% and an RR reduction of 61.57% in terms of the adverse reactions. Based on the TSA, the required information size (RIS) was determined as 168. The cumulative Z-curve surpassed the adjusted boundary of the RIS, indicating that ZGCD therapy would yield definitive and strong outcomes (Figure 10B).

## 4 Results of network pharmacology

### 4.1 Analysis of ZGCD and DCM targets

**Figure 11:**
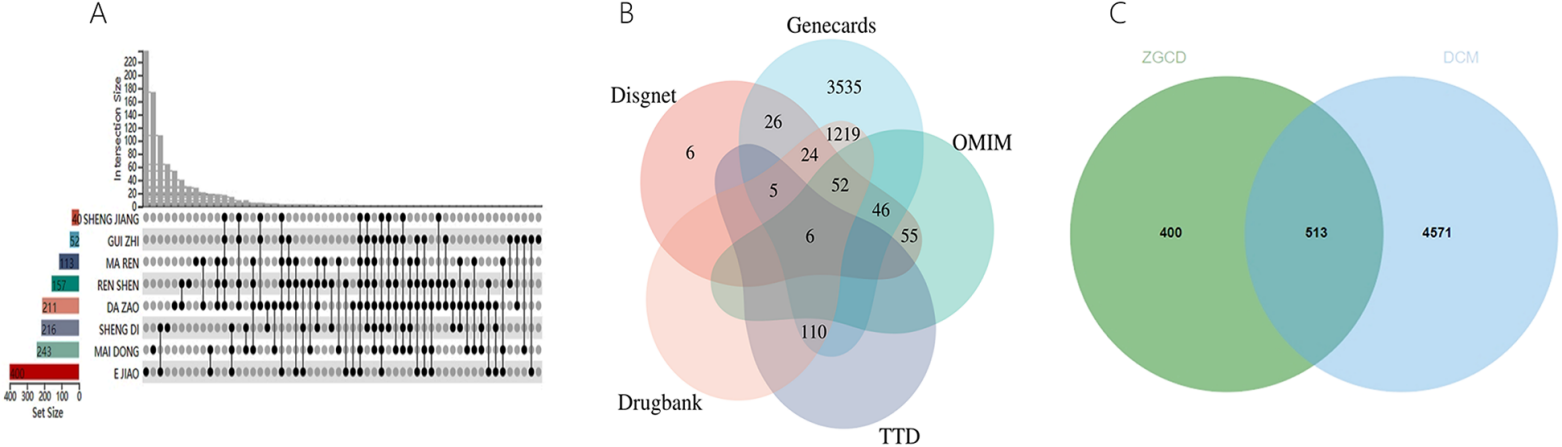
**(A)** Gene targets of ZGCD, **(B)** multiple databases of disease targets for DCM, **(C)** venn graph of intersection targets.

A combined total of 66 ZGCD-related active ingredients and 913 corresponding targets were gathered from the TCMSP and BATMAN-TCM databases. Additionally, 5,084 targets associated with DCM were identified through the Drugbank, OMIM, TTD, DisGeNET and GeneCards databases. The overlap of drug and DCM targets yielded a total of 513 shared targets, as illustrated in the Venn diagram (Figure 11 and Supplementary Material S4).

### 4.2 The construction of the H-C-T network

**Figure 12:**
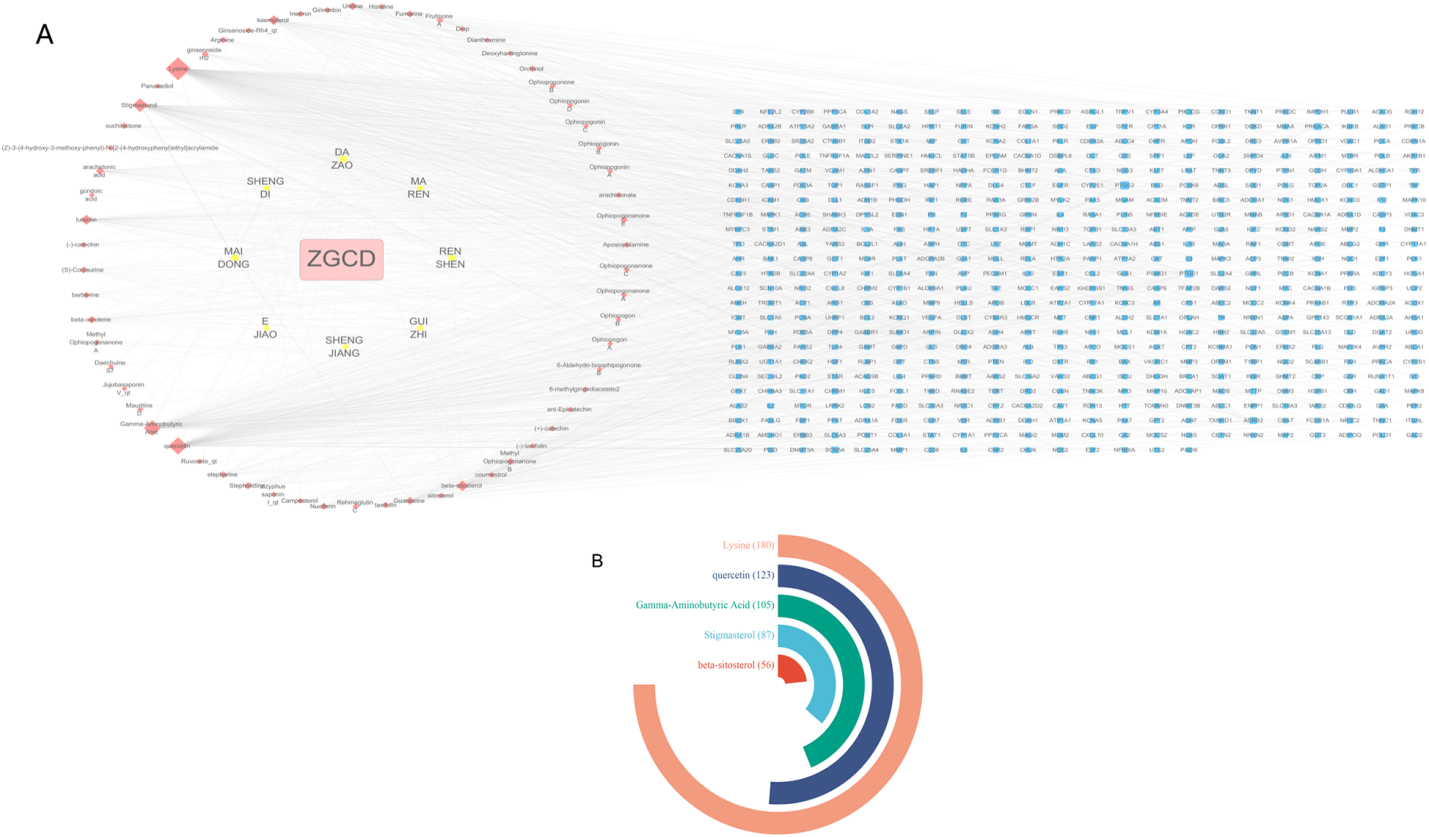
**(A)** Herb-compound-target network of ZGCD for DCM; **(B)** Top five degree of the compounds.

A total of 591 nodes and 1,110 edges comprising 8 herbs, 62 compounds, and 521 genes made up the Herb-Compound-Target (H-C-T) network (Figure 12A). Statistics indicated that nodes with a larger size are more significant. The top five compounds, as determined by degree analysis, were lysine (180), quercetin (123), gamma- aminobutyric acid (105), stigmasterol (87), and beta-sitosterol (Figure 12B) (56). Detailed information was presented in Table 3 and Supplementary Material S5.

**Table 3:** Compounds of ZGCD.

Molecular name	Degree	Molecular name	Degree	Molecular name	Degree
Lysine	180	berberine	12	Mauritine D	3
quercetin	123	suchilactone	12	Diop	3
Gamma-Aminobutyric Acid	105	Arginine	11	Dianthramine	3
Stigmasterol	87	Inermin	10	Deoxyharringtonine	3
Beta-sitosterol	56	taxifolin	9	arachidonate	3
Luteolin	48	Girinimbin	8	sitosterol	3
Kaempferol	47	Aposiopolamine	8	(-)-taxifolin	3
Uridine	28	Rehmaglutin C	7	Ophiopogonin D	2
Guanosine	28	coumestrol	7	Ophiopogonin C	2
Arachidonic acid	26	(-)-catechin	7	Ophiopogonin B	2
Beta-carotene	23	(+)-catechin	7	Ophiopogonin A	2
Stepholidine	21	Ophiopogonanone A	6	Ophiopogon B	2
Fumarine	20	Methyl Ophiopogonanone A	6	Ophiopogon A	2
Nuciferin	19	(Z)-3-(4-hydroxy-3-methoxy-phenyl)-N-[2-(4-hydroxyphenyl)ethyl]acrylamide	6	zizyphus saponin I_qt	2
Ophiopogonanone E	17	Ophiopogonanone C	5	Ruvoside_qt	2
Methyl Ophiopogonanone B	17	Campesterol	5	Jujubasaponin V_qt	2
stepharine	17	ent-Epicatechin	5	Daechuine S7	2
(S)-Coclaurine	17	Histidine	4	gondoic acid	2
ginsenoside rh2	13	Ophiopogonone B	4	Panaxadiol	2
Frutinone A	13	6-methylgingediacetate2	4	Ginsenoside-Rh4_qt	2
Orchinol	12	6-Aldehydo-Isoophipogonone B	3		

### 4.3 Protein-protein interaction network analysis

**Figure 13:**
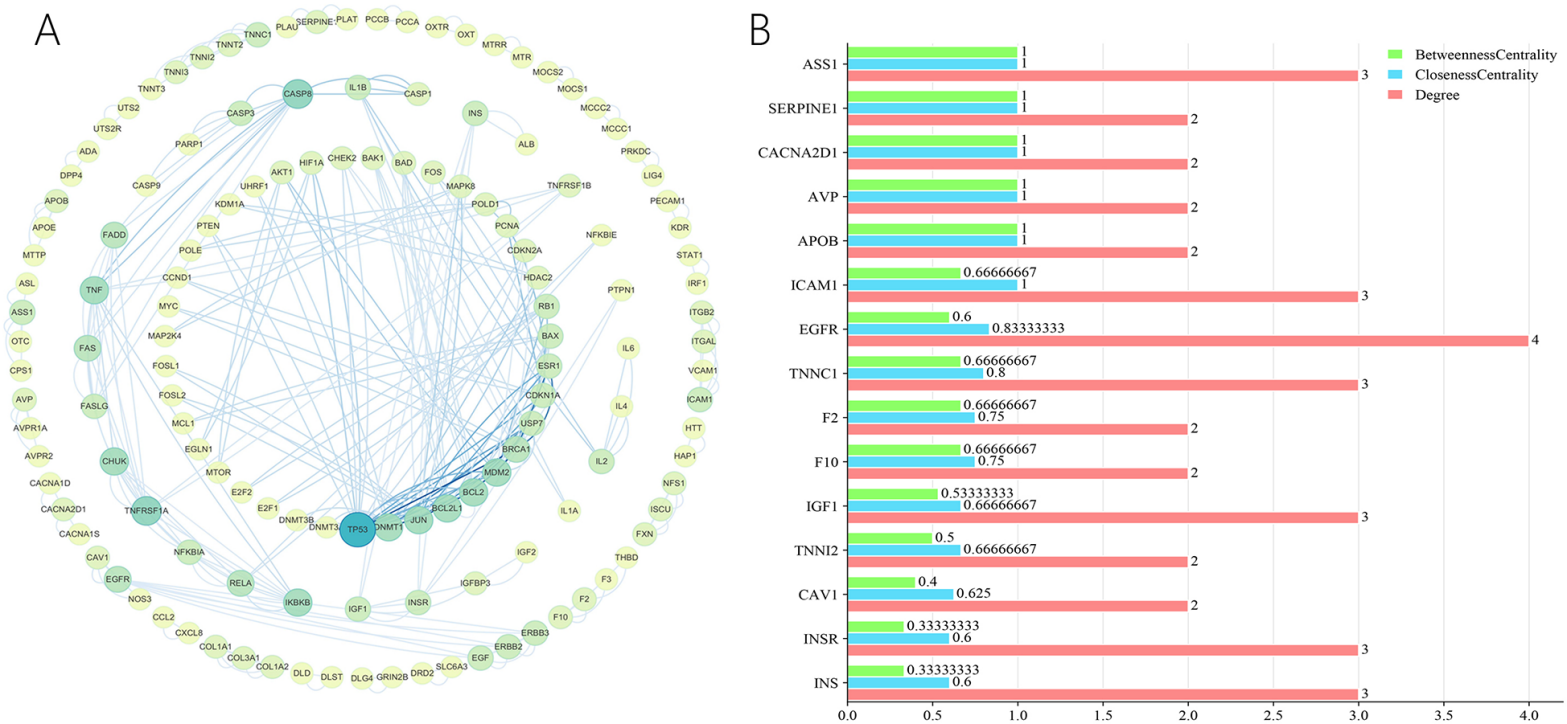
**(A)** The PPI network of the intersection genes of herb targets and disease targets; **(B)** the core genes from the intersection genes.

The STRING platform was used for the analysis of protein-protein interactions. In total, 513 predicted targets were entered and interactions with a confidence score over 0.9 were chosen for further analysis. A PPI network was subsequently constructed by eliminating isolated nodes and visualizing the resultant targets using Cytoscape. The network contains 138 nodes and 274 edges, as shown in Figure 13A. Additionally, topological analysis was performed based on BC, CC, and DC to identify potential key targets for ZGCD in managing DCM. Ultimately, 15 core targets were obtained, including ASS1, SERPINE1, CACNA2D1, AVP, APOB, ICAM1, EGFR, TNNC1, F2, F10, IGF1, TNNI2, CAV1, INSR, INS, as shown in Figure 13B. Detailed information was presented in Table 4 and Supplementary Material S6.

**Table 4:** Detail of core targets.

Gene	Betweenness centrality	Closeness centrality	Degree
ASS1	1	1	3
SERPINE1	1	1	2
CACNA2D1	1	1	2
AVP	1	1	2
APOB	1	1	2
ICAM1	0.66666667	1	3
EGFR	0.6	0.83333333	4
TNNC1	0.66666667	0.8	3
F2	0.66666667	0.75	2
F10	0.66666667	0.75	2
IGF1	0.53333333	0.66666667	3
TNNI2	0.5	0.66666667	2
CAV1	0.4	0.625	2
INSR	0.33333333	0.6	3
INS	0.33333333	0.6	3

### 4.4 GO and KEGG enrichment analysis

**Figure 14:**
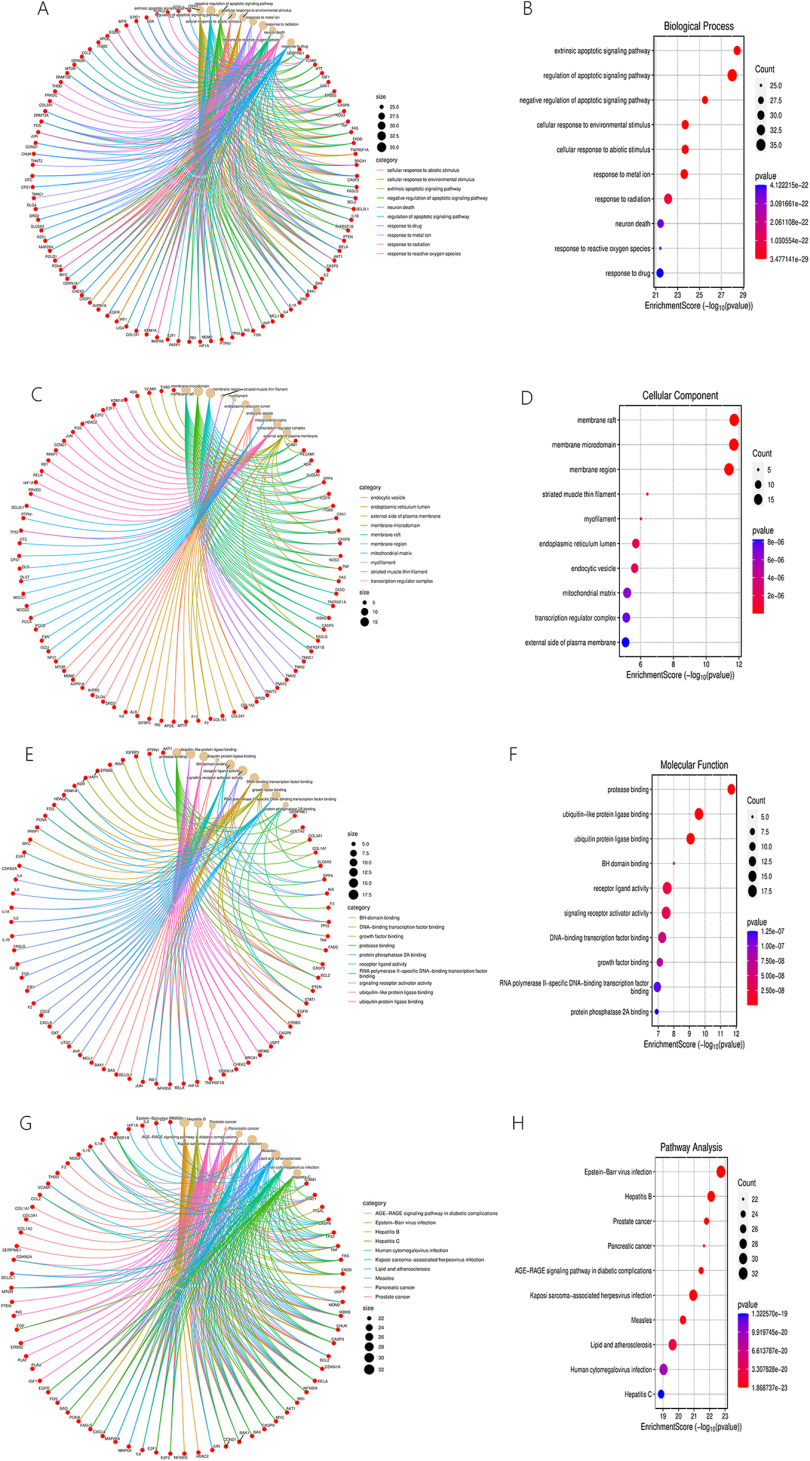
BP **(A,B)**, CC **(C,D)**, MF **(E,F)** categories of GO enrichment analysis and results of KEGG enrichment analysis **(G,H)**.

As part of GO enrichment analysis, Figures 14A–F shows the top ten significant terms in the biological process (BP), cellular component (CC) and molecular function (MF) categories. The BP category mainly included but not limited to regulation of apoptotic signaling pathway, response to reactive oxygen species. The CC category mainly but not limited to membrane raft, myofilament, and endoplasmic reticulum lumen. The MF category mainly included but not limited to protease binding, and signaling receptor activator activity. Detailed information was presented in and Supplementary Material S7.

A visual representation of the top 10 items from the KEGG enrichment analysis is shown in Figures 14G,H. These results mostly concerned the Advanced Glycation End Products (AGEs)-Receptor for Advanced Glycation End Products (RAGE) signaling pathway in diabetic complications, lipids and atherosclerosis, etc. Detailed information was presented in and Supplementary Material S7.

## 5 Discussion

There is a wide range of abnormalities contributing to the pathophysiology of diabetic cardiomyopathy, including but not limited to glucotoxicity, cardiac insulin resistance, oxidative stress, impaired calcium handling, activation of the renin-angiotensin-aldosterone system, mitochondrial dysfunction, etc. Despite some progress in understanding the pathogenesis of diabetic cardiomyopathy, the availability of effective therapeutic interventions targeting this condition remains limited, which may be attributed to the presence of treatment resistance or adverse reactions in a subset of patients to conventional therapies. ZGCD, derived from “Treatise on Febrile Diseases,” exerts a notable impact on a variety of cardiovascular diseases. Nevertheless, the intricate nature of its constituents has hindered a comprehensive elucidation of its mechanism of action. Findings from the current investigation suggest that the efficacy of ZGCD in treating DCM is markedly superior when used in isolation or in conjunction with western medications, as opposed to western medications alone. Furthermore, the alleviation of symptoms related to diminished cardiac function and hypertension in DCM patients was significantly enhanced using ZGCD, with a lower incidence of adverse reactions. Meanwhile, the analysis revealed that certain compounds within ZGCD and its primary targets were associated with anti-DCM activity.

### 5.1 Summary of the meta-analysis findings

A meta-analysis encompassing 9 studies meeting the specified inclusion criteria was undertaken, involving a total of 661 participants. Overall, ZGCD therapy exhibited a higher total effective rate for DCM compared to treatment with western medicine alone. Moreover, LVEDV, LVSDV, LVEF, and LVDD are commonly utilized echocardiographic parameters that characterize ventricular systolic function and its remodeling (36). For the purpose of assessing cardiovascular outcomes, we evaluated all above-mentioned indicators as a composite measure. Our findings confirmed that the use of ZGCD therapy, as opposed to WM, showed benefits in enhancing cardiac function indicators. Moreover, blood pressure are commonly measured indicators of cardiovascular functioning (37). DBP and SBP were therefore our choice for assessing cardiac function. According to our results, the ZGCD therapy resulted in meaningful reductions of blood pressure, indicating that improvements in cardiovascular function can be achieved with ZGCD use. As for safety, there were significantly fewer adverse reactions observed in the ZGCD groups than in the control group, indicating a favorable safety profile.

### 5.2 Summary of the network pharmacology findings

To further explore the potential mechanisms underlying the anti-DCM effects of ZGCD, network pharmacology analysis was conducted. A total of 138 core intersection targets were identified from a pool of 5084 DCM-related targets and 913 targets associated with the components of ZGCD. The top five active ingredients identified in the H-C-T network were lysine, quercetin, gamma-aminobutyric acid, stigmasterol, and beta-sitosterol. Lysine, an essential amino acid known for its role in growth promotion, immune system support, and metabolic processes, emerged as a key player in the potential therapeutic effects of ZGCD on DCM. Extensive analysis of metabolites in large-scale prospective cohort studies has revealed a correlation between lysine levels and decreased mortality risk in individuals with T2DM and cardiovascular conditions (38). Additionally, reduced lysine acetylation has been shown to mitigate metabolic inflexibility and cardiac dysfunction in diabetic hearts through the utilization of mitochondrial alternative electron carriers, suggesting a promising therapeutic strategy for diabetes (39). Quercetin, a flavonoid found in a variety of foods and plants, is widely distributed. A recent study demonstrated that quercitrin has the potential to mitigate high glucose-induced cellular pyroptosis activation and mitochondrial damage through the Nrf2 signaling pathway, thereby offering protection against structural damage and morphological alterations in cardiomyocytes of diabetic rats (40). Likewise, quercetin was observed to prevent excessive cellular apoptosis of cardiomyocytes by reducing the expressions of caspase 9 and caspase 3 in diabetic rats (41). GABA signaling has been demonstrated to attenuate Arx expression for facilitating insulin expression in adult pancreatic α- and duct-cells (42). The exogenous administration of GABA significantly enhanced *α*-cell transdifferentiation towards a β-cell phenotype, decreased circulating glucagon levels, and improved pancreatic insulin stores in STZ-diabetic mice lacking insulin (43). Stigmasterol and β-sitosterol exhibit diverse pharmacological effects, such as potent *in vivo* antioxidant activity, anti-apoptotic properties, cell cycle modulation, anti-mutagenic effects, inhibition of angiogenesis, and anti-inflammatory properties. Specifically, these compounds were able to restore the impaired functionality of pancreatic β cells, thereby regulating serum glucose levels by tightly controlling insulin secretion (44). Furthermore, stigmasterol demonstrated anti-inflammatory effects through glucocorticoid-mediated reduction in neutrophilic leukocyte infiltration and alleviated edema symptoms in the later stages of DCM by decreasing arachidonic acid and its metabolites (45).

Next, according to the PPI network analyses, ASS1, SERPINE1, CACNA2D1, AVP, APOB, ICAM1, EGFR, TNNC1, F2, F10, IGF1, TNNI2, CAV1, INSR and INS were potential anti-DCM targets of ZGCD. ASS-1, a crucial enzyme in arginine metabolism, has been shown to potentially promote islet neogenesis and β cell proliferation through the activation of mTOR signaling, as indicated by existing research (46). Moreover, the restoration of ASS-1 expression has been shown to enhance the production of nitric oxide, leading to improved injuries of the coronary artery endothelium in STZ-induced diabetic rats (47). SERPINE1, a member of the Serine protease inhibitor family, plays a crucial role in modulating the plasminogen/plasminase system (48). Previous studies utilizing single-cell sequencing technology have revealed an upregulation of SERPINE1 in myocardial tissue of STZ-induced diabetic mice, suggesting that SERPINE1 may serve as an independent pathogenic trigger of diabetic cardiomyopathy (49). A significant role is played by CACNA2D1 in calcium channel regulation (50), with documented effects on ventricular arrhythmia syndromes, neurotransmitter release, muscle contraction, and neural signal transduction (51, 52). AVP is produced by magnocellular neurons situated in the supraoptic and paraventricular nuclei of the hypothalamus, and is subsequently stored in the posterior pituitary gland. Apart from its function as a neuroendocrine messenger, AVP is associated with the progression of myocardial remodeling and the facilitation of water-sodium retention in the early stages of heart failure (53, 54). APOB acts as the primary structural protein in lipoprotein particles secreted from cells. A study in STZ-induced diabetic mice demonstrated that overexpressing the APOB transgene in the heart could reduce cardiac lipid accumulation and alter markers of cardiac dysfunction (55). EGFR has the ability to translate external stimuli into internal signals upon activation, playing a significant role in cell differentiation and proliferation (56). Prior research has established that downregulating EGFR protein expression in myocardial tissue effectively inhibits the onset and advancement of diabetic cardiomyopathy (57). TNNC1 serves as a crucial structural element in upholding myofibril integrity, potentially causing disruptions in Ca2+ cycling mechanisms and triggering compensatory cardiomyocyte hypertrophy, ultimately culminating in the progression of hypertrophic cardiomyopathy (58). The accelerated degradation of the TNNC1 protein in the myocardium of T2DM rats is believed to contribute to the disruption of myofibrillar ganglion structure, myofibrillar rupture, and dissolution in T2DM rat cardiomyocytes (59). TNNI3, a human inhibitory cardiac troponin, plays a role in safeguarding cardiac function by activating a self-protective response (60). IGF-1, reducing hepatic glucose production and promoting peripheral glucose uptake, plays a key role in maintaining glucose homeostasis (61). Studies have shown that IGF-1 can effectively improve diabetic cardiomyopathy through its antioxidant and anti-inflammatory effects, as well as by activating the Akt/GSK-3β signaling pathway (62). Coagulation factors, such as F2 and F10, play a crucial role in various physiological processes including apoptosis, angiogenesis, and inflammatory responses. Zheng's research demonstrated that silencing the F2 gene can inhibit apoptosis and improve heart function in streptozotocin-induced diabetic rats (63). CAV1 serves as a key structural component of caveolae, specialized plasma membrane invaginations involved in molecular transport, cell adhesion, and signal transduction (64). Dysregulation of CAV1 protein expression can lead to abnormal nitric oxide levels in the cardiovascular system and disrupt Ca2+ homeostasis (65, 66). INS and INSR play a role in promoting the synthesis of glycogen, lipid, and protein, resulting in a hypoglycemic effect and enhancing energy uptake and utilization in cardiomyocytes, thereby maintaining their structure and function (67). This research serves as a pharmacological foundation for the clinical effectiveness of ZGCD in treating DCM. While the impact of ZGCD on DCM has been demonstrated through its components, the intricate nature of these components has prevented a clear understanding of the specific mechanisms involved. Therefore, this study aims to investigate the pathways through which ZGCD may exert its therapeutic effects on DCM using KEGG and GO enrichment analyses.

The current enrichment analysis of ZGCD on DCM has identified the AGEs-RAGE signal pathway, lipid and atherosclerosis as significant mechanisms. The AGEs-RAGE signal pathway is increasingly recognized as a potential therapeutic target for DCM. In a high-glucose environment, AGEs can bind to the AGE receptor, leading to the activation of its signaling pathway. This activation regulates oxidative stress, inflammatory reactions, and endothelial cell proliferation, ultimately contributing to the initiation and progression of diabetic cardiomyopathy (68–70). Diabetic rats exhibit elevated levels of AGEs and increased expression of RAGEs in myocardial tissues as the disease progresses, along with worsening fibrosis (71). Lipid metabolism disorder is a prominent metabolic alteration observed in DCM. Diabetic rats also demonstrate heightened fatty acid influx, leading to lipid accumulation and cardiac lipotoxicity, contributing to the development of DCM (72). Furthermore, DCM is characterized by increased stiffness in the atrial and ventricular walls due to arteriosclerosis (73). In summary, the aforementioned findings suggest that ZGCD may serve as a safe and effective treatment for DCM through multiple pathways and targets.

### 5.3 Research on possible mechanisms

During recent years, there has been an intense interesting on the therapeutic mechanisms of ZGCD on DCM. The existing evidence suggestes that the underlying mechanisms have a tight relationship with autophagy and miR-181a-5p. The autophagic process plays a critical regulatory role in multiple key pathways implicated in diabetic cardiomyopathy, encompassing myocardial cell lipotoxicity stemming from aberrant glucose metabolism or elevated serum free fatty acids, as well as myocardial dysfunction and cardiac structural alterations arising from oxidative stress or insulin resistance (74). Concurrently, diabetes precipitates energy deficits and dysregulated lipid metabolism, culminating in the generation of numerous lipotoxic intermediates that heighten myocardial cell apoptosis (75). Hu et al. discovered that Zhigancao Decoction was able to decrease the serum BNP level in rats with DCM, enhance EF, and ameliorate cardiac function in this model. Additionally, In the DCM model rats, researchers noted reduced Bcl-2 expression, elevated Bax expression, decreased LC3-II/LC3-I and beclin-1 expression, as well as increased p62 expression (76). Hence, it is hypothesized that Zhigancao Decoction may augment the autophagic activity of myocardial cells, thereby enhancing their capacity to eliminate dysfunctional organelles, enhance energy metabolism, suppress apoptosis, and ultimately ameliorate cardiac function in rats with dilated cardiomyopathy.

MiR-181a-5p is a versatile microRNA that exerts significant regulatory influence over numerous physiological and pathological processes. Inhibition of miR-181a-5p has been shown to upregulate PDCD4 expression, subsequently enhancing apoptosis in DCM cardiomyocytes (77). SPHK2, a multifunctional lipid kinase, is ubiquitously distributed across multiple organelles and governs numerous crucial molecular pathways (78). Wang et al. discovered that in DCM rats, the myocardial expression of miR181a-5p was notably reduced and the protein levels of SPHK2 were significantly elevated compared to normal rats. Following treatment with ZGCD, there was a reversal in these trends, with an increase in miR-181a-5p expression and a decrease in SPHK2 protein levels. Subsequent cell experiments revealed that high glucose-induced cardiac fibroblasts exhibited down-regulated miR-181a-5p expression and up-regulated SPHK2 protein expression (79). Following pretreatment with ZGCD, miR-181a-5p expression levels increased. These findings ultimately support the notion that ZGCD can effectively regulate myocardial fibrosis and improve diabetic cardiomyopathy via targeting SPHK2 with miR-181a-5p.

Taken together, autophagy, and microRNA might be the underlying pathogenesis of DCD.

### 5.4 Advantages and limitations

The meta-analysis part of our study represents the initial attempt to quantitatively assess the advantageous impact of ZGCD therapy on DCM, offering a more robust evidence-based assessment for researchers both domestically and internationally. Adhering to the guidelines set forth by the Cochrane Collaboration, the primary aim is to generate more thorough and conclusive findings. The incorporation of supplementary outcome measures, such as cardiac function indicators, blood pressure and safety indicators, facilitates a comprehensive and layered evaluation of the efficacy of ZGCD in the treatment of DCM. Meanwhile, this study represents the initial attempt to combine meta-analysis and network pharmacology in assessing the effectiveness and potential mechanisms of ZGCD in individuals with DCM. The findings of this study have contributed to advancing knowledge in this area and have provided direction for further research.

However, it is important to acknowledge certain potential limitations despite our thorough assessment of the existing evidence. Firstly, concerning the research methodology, the studies incorporated in the meta-analysis may evidently lack rigor in terms of randomization procedures, allocation concealment, and blinding. For instance, the implementation of blinding poses a challenge when both herbal decoctions and WM are used as interventions, due to their distinct external characteristics. Additionally, network pharmacology partially relies on drug-likeness and oral bioavailability values to screen herbal ingredients, potentially overlooking some active components. These factors may compromise the methodological rigor of the research, thereby diminishing the reliability and credibility of the findings. Secondly, regarding potential biases, the small sample size in most studies included in our analysis may lead to an overestimation of the intervention effects and introduce bias. Moreover, the presence of various confounding variables, such as different WM interventions in the control group, may also contribute to potential biases. Furthermore, the variability in medical environments across different regions where studies are conducted introduces a significant likelihood of discrepancies in the measurement tools and evaluation methods employed. Such inconsistencies may result in potential biases, adversely affecting the interpretation and comparison of the findings. Thirdly, it is essential to note that each of the identified articles included in the review was published in Chinese and was conducted in China. Therefore, it is essential to validate our results to determine the generalizability of TCM in larger and more diverse populations across different countries and ethnicities. Fourthly, a paucity of research is available on the follow-up data of ZGCD in DCM management. Considering DCM's chronic and progressive nature, which can fluctuate over time, continuous follow-up assessments are essential for assessing its true effectiveness and long-term effectiveness. Fifthly, it is important to note that this study is primarily based on literature reviews and database analyses, therefore, further experiments and clinical trials are necessary to validate the specific conclusions drawn.

### 5.5 Implications for clinical practice and future research

According to our findings, the therapy mentioned for DCM is effective and safe, which provides a solid basis for developing clinical guidelines. Detailed examination of ZGCD therapy in DCM contributes greatly to preserving TCM clinical wisdom, enhancing understanding of TCM theory and practice in DCM treatment, and providing more treatment options.

Based on our results and the constraints identified, the following recommendations have been made for future studies and clinical applications. First of all, for the results to be more generalizable and valid, multicenter studies with large samples are recommended. Secondly, it is imperative for research related to TCM to improve its methodologies by emphasizing rigorous protocols and quality control measures, including the meticulous implementation of randomization, blinding as well as allocation concealment. Thirdly, for extended research and clinical trials, an adequate follow-up period tailored to the disease's specific characteristics is essential to guarantee the long-term safety of ZGCD in DCM patients, assess the most effective dosage and treatment duration, and detect any potential adverse events, thus offering valuable insights for clinical practice. Fourthly, investigating the research possibilities associated with utilizing Chinese herbal medicines for the management of DM and its complications, conducting pre-clinical and clinical trials focusing on the active constituents of herbal medicines may offer valuable insights into the precise therapeutic effects and underlying mechanisms of treating DCM. This approach could strengthen the existing evidence base supporting the utilization of Chinese herbal medicines in DCM and facilitate the incorporation of TCM into relevant international guidelines. In future research endeavors, it is imperative to prioritize the advancement of high-quality clinical trial data concerning ZGCD in the treatment of DCM, especially randomized controlled trials, in order to substantiate the hypotheses derived from meta-analyses and network pharmacological analyses.

The integration of TCM with WM has been demonstrated to enhance treatment efficacy for numerous diseases, such as T2DM, Non-Alcoholic Fatty Liver Disease (NAFLD), hypertension, and even COVID-19, as supported by a growing body of evidence-based practices. The Chinese medical community has recognized the distinct characteristics and functions of TCM and WM, striving to synthesize the most effective practices from both systems. Specifically, in terms of diagnosis, the combination of WM diagnostic methods with pulse and tongue examination, the unique examination techniques of TCM, allows for a more comprehensive understanding of diseases and facilitates the development of more rational treatment plans. Regarding treatment methodologies, integrating WM with TCM syndrome differentiation can provide more precise therapeutic approaches, enhancing treatment outcomes by addressing the different stages and characteristics of diseases. Furthermore, for individuals experiencing significant adverse reactions to WM treatments, the integration of TCM with WM may mitigate the occurrence of such adverse reactions while preserving therapeutic efficacy, thereby offering an alternative treatment option.

## 6 Conclusion

This study analyzed the effectiveness and mechanism of ZGCD in the treatment of DCM. Our findings revealed that compared to western medicine alone, the utilization of ZGCD had better clinical efficacy and fewer side effects, especially in mitigating blood pressure and enhancing cardiac function. Five components of ZGCD, including lysine, quercetin, gamma- aminobutyric acid, stigmasterol, and beta-sitosterol, may play anti-DCM roles via the targets of ASS1, SERPINE1, CACNA2D1, AVP, APOB, ICAM1, EGFR, TNNC1, F2, F10, IGF1, TNNI2, CAV1, INSR and INS. The AGEs/RAGE signaling pathway, as well as lipid and atherosclerosis may be primary mechanism for ZGCD to achieve its anti-DCM effect.

## Data Availability

The original contributions presented in the study are included in the article/Supplementary Material, further inquiries can be directed to the corresponding authors.
