# Supplementary material for: Integrated meta-analysis and network pharmacology analysis: evaluation of Zhigancao decoction as treatment for diabetic cardiomyopathy
Source: Front Cardiovasc Med. 2025 Mar 14;12:1454647. doi: 10.3389/fcvm.2025.1454647 (PMC11949964; doi:10.3389/fcvm.2025.1454647)
Supplement: Supplementary file 1 [file Datasheet1.docx]

**Details of Search Strategy**

**Source: PubMed**

| Search | Query |
| --- | --- |
| #1 | “Diabetic Cardiomyopathy” [Mesh] |
| #2 | Diabetic Cardiomyopathy [Title/Abstract] |
| #3 | Diabetes-associated Cardiomyopathy [Title/Abstract] |
| #4 | Diabetic Cardiovascular Disorder [Title/Abstract] |
| #5 | Diabetes-associated Cardiovascular Disorder [Title/Abstract] |
| #6 | Diabetic Cardiovascular Outcome [Title/Abstract] |
| #7 | Diabetes-associated Cardiovascular Outcome [Title/Abstract] |
| #8 | #1 OR #2 OR #3 OR #4 OR #5OR #6OR #7 |
| #9 | Zhigancao Decoction [Title/Abstract] |
| #10 | Zhigancao Soup [Title/Abstract] |
| #11 | Zhigancao Tang [Title/Abstract] |
| #12 | Fumai Decoction [Title/Abstract] |
| #13 | Fumai Soup [Title/Abstract] |
| #14 | Fumai Tang[Title/Abstract] |
| #15 | Chinese herbal medicine [Title/Abstract] |
| #16 | Chinese traditional [Title/Abstract] |
| #17 | Oriental traditional [Title/Abstract] |
| #18 | Traditional Chinese medicine [Title/Abstract] |
| #19 | Traditional Chinese medicinal materials [Title/Abstract] |
| #20 | Chinese herb* [Title/Abstract] |
| #21 | Herbal medicine [Title/Abstract] |
| #22 | Herbal decoction [Title/Abstract] |
| #23 | #9 OR #10 OR #11 OR #12 OR #13 OR #14 OR #15 OR #16 OR #17 OR #18 OR #19 OR #20 OR #21 OR #22 |
| #24 | Controlled trial [Title/Abstract] |
| #25 | CT [Title/Abstract] |
| #26 | Clinical trial [Title/Abstract] |
| #27 | Placebo [Title/Abstract] |
| #28 | #24 OR #25 OR #26 OR #27 |
| #29 | #1 AND #23 AND #28 |

**Source: Cochrane Library**

| Search | Query |
| --- | --- |
| #1 | MeSH descriptor: [Diabetic Cardiomyopathy] explode all trees |
| #2 | (Diabetic Cardiomyopathy): ti,ab,kw |
| #3 | (Diabetes-associated Cardiomyopathy): ti,ab,kw |
| #4 | (Diabetic Cardiovascular Disorder): ti,ab,kw |
| #5 | (Diabetes-associated Cardiovascular Disorder): ti,ab,kw |
| #6 | (Diabetic Cardiovascular Outcome): ti,ab,kw |
| #7 | (Diabetes-associated Cardiovascular Outcome): ti,ab,kw |
| #8 | #1 OR #2 OR #3 OR #4 OR #5OR #6OR #7 |
| #9 | (Zhigancao Decoction): ti,ab,kw |
| #10 | (Zhigancao Soup): ti,ab,kw |
| #11 | (Zhigancao Tang): ti,ab,kw |
| #12 | (Fumai Decoction): ti,ab,kw |
| #13 | (Fumai Soup): ti,ab,kw |
| #14 | (Fumai Tang): ti,ab,kw |
| #15 | (Chinese herbal medicine): ti,ab,kw |
| #16 | (Chinese traditional): ti,ab,kw |
| #17 | (Oriental traditional): ti,ab,kw |
| #18 | (Traditional Chinese medicine): ti,ab,kw |
| #19 | (Traditional Chinese medicinal materials): ti,ab,kw |
| #20 | (Chinese herb): ti,ab,kw |
| #21 | (Herbal medicine): ti,ab,kw |
| #22 | (Herbal decoction): ti,ab,kw |
| #23 | #9 OR #10 OR #11 OR #12 OR #13 OR #14 OR #15 OR #16 OR #17 OR #18 OR #19 OR #20 OR #21 OR #22 |
| #24 | (Controlled trial): ti,ab,kw |
| #25 | (CT): ti,ab,kw |
| #26 | (Clinical trial): ti,ab,kw |
| #27 | (Placebo): ti,ab,kw |
| #28 | #24 OR #25 OR #26 OR #27 |
| #29 | #1 AND #23 AND #28 |

**Source: Embase**

| Search | Query |
| --- | --- |
| #1 | 'Diabetic Cardiomyopathy '/exp |
| #2 | 'Diabetic Cardiomyopathy': ab, ti |
| #3 | 'Diabetes-associated Cardiomyopathy': ab, ti |
| #4 | 'Diabetic Cardiovascular Disorder': ab, ti |
| #5 | 'Diabetes-associated Cardiovascular Disorder': ab, ti |
| #6 | 'Diabetic Cardiovascular Outcome': ab, ti |
| #7 | 'Diabetes-associated Cardiovascular Outcome': ab, ti |
| #8 | #1 OR #2 OR #3 OR #4 OR #5OR #6OR #7 |
| #9 | 'Zhigancao Decoction': ab, ti |
| #10 | 'Zhigancao Soup': ab, ti |
| #11 | 'Zhigancao Tang': ab, ti |
| #12 | 'Fumai Decoction': ab, ti |
| #13 | 'Fumai Soup': ab, ti |
| #14 | 'Fumai Tang': ab, ti |
| #15 | 'Chinese herbal medicine': ab, ti |
| #16 | 'Chinese traditional': ab, ti |
| #17 | 'Oriental traditional': ab, ti |
| #18 | 'Traditional Chinese medicine': ab, ti |
| #19 | 'Traditional Chinese medicinal materials': ab, ti |
| #20 | 'Chinese herb': ab, ti |
| #21 | 'Herbal medicine': ab, ti |
| #22 | 'Herbal decoction': ab, ti |
| #23 | #9 OR #10 OR #11 OR #12 OR #13 OR #14 OR #15 OR #16 OR #17 OR #18 OR #19 OR #20 OR #21 OR #22 |
| #24 | 'Controlled trial': ab, ti |
| #25 | 'CT': ab, ti |
| #26 | 'Clinical trial': ab, ti |
| #27 | 'Placebo': ab, ti |
| #28 | #24 OR #25 OR #26 OR #27 |
| #29 | #1 AND #23 AND #28 |

**Source: Web of Science**

| Search | Query |
| --- | --- |
| #1 | TS="Diabetic Cardiomyopathy" |
| #2 | TS="Diabetes-associated Cardiomyopathy" |
| #3 | TS="Diabetic Cardiovascular Disorder" |
| #4 | TS="Diabetes-associated Cardiovascular Disorder" |
| #5 | TS="Diabetic Cardiovascular Outcome" |
| #6 | TS="Diabetic Cardiovascular Outcome" |
| #7 | TS="Diabetes-associated Cardiovascular Outcome" |
| #8 | #1 OR #2 OR #3 OR #4 OR #5OR #6OR #7 |
| #9 | TS="Zhigancao Decoction" |
| #10 | TS="Zhigancao Soup" |
| #11 | TS="Zhigancao Tang" |
| #12 | TS="Fumai Decoction" |
| #13 | TS="Fumai Soup" |
| #14 | TS="Fumai Tang" |
| #15 | TS="Chinese herbal medicine" |
| #16 | TS="Chinese traditional" |
| #17 | TS="Oriental traditional" |
| #18 | TS="Traditional Chinese medicine" |
| #19 | TS="Traditional Chinese medicinal materials" |
| #20 | TS="Chinese herb" |
| #21 | TS="Herbal medicine" |
| #22 | TS="Herbal decoction" |
| #23 | #9 OR #10 OR #11 OR #12 OR #13 OR #14 OR #15 OR #16 OR #17 OR #18 OR #19 OR #20 OR #21 OR #22 |
| #24 | TS="Controlled trial" |
| #25 | TS="CT" |
| #26 | TS="Clinical trial" |
| #27 | TS="Placebo" |
| #28 | #24 OR #25 OR #26 OR #27 |
| #29 | #1 AND #23 AND #28 |

**CNKI**

(SU = '糖尿病相关心肌病' OR SU='糖尿病相关心肌损害' ) AND ( FT='中医' OR FT='中药' OR FT='中医药' OR FT='中西医' OR FT='中成药' OR FT='炙甘草汤' OR FT='复脉汤' OR FT='片' OR FT='丸' OR FT='散' OR FT='胶囊' OR FT='颗粒' OR FT='水' OR FT='液' OR FT='合剂' OR FT='注射液') AND (FT='对照研究'OR FT='临床研究'OR FT='疗效观察')

**VIP**

(M=糖尿病相关心肌病 OR 糖尿病相关心肌损害) AND ((U=炙甘草汤 OR 复脉汤 OR 中医 OR 中药 OR 中西医OR中成药 OR 汤 OR 片 OR 丸 OR 散 OR 胶囊 OR 颗粒 OR水 OR液 OR 合剂 OR 注射液 ) OR (R=炙甘草汤 OR 复脉汤 OR 中医 OR 中药 OR 中西医OR中成药 OR 汤 OR 片 OR 丸 OR 散 OR 胶囊 OR 颗粒 OR水 OR液 OR 合剂 OR 注射液)) AND ((U=对照研究 OR 临床研究 OR 疗效观察) OR (R=对照研究 OR 临床研究 OR 疗效观察))

**Wanfang**

全部:(( "糖尿病相关心肌病" OR "糖尿病相关心肌损害") AND ( "炙甘草汤" OR "复脉汤" OR "中医" OR "中药" OR "中西医" OR "中成药" OR "汤" OR "片" OR "丸" OR "散" OR "胶囊" OR "颗粒" OR "水" OR "液" OR "合剂" OR "注射液" ) AND ("对照研究" OR "临床研究" OR "疗效观察" )

**CBM**

(("糖尿病相关心肌病"[全部字段:智能] OR "糖尿病相关心肌损害"[全部字段:智能] )) AND(("炙甘草汤"[全部字段:智能] OR"复脉汤"[全部字段:智能] OR "中医"[全部字段:智能] OR "中药"[全部字段:智能] OR "中西医"[全部字段: 智能] OR ("中成药"[常用字段:智能] OR "汤"[全部字段:智能]) OR "片"[全部字段:智能] OR "丸"[全部字段:智能] OR "散"[全部字段:智能] OR "胶囊"[全部字段:智能] OR "颗粒"[全部字段:智能] OR "水"[全部字段:智能] OR"液"[全部字段:智能] OR "合剂"[全部字段:智能] OR "注射液"[全部字段:智能])) AND ("对照研究"[全部字段:智能] OR "临床研究"[全部字段:智能] OR "疗效观察"[全部字段:智能])
