# Supplementary material for: Integrated meta-analysis and network pharmacology analysis: evaluation of Zhigancao decoction as treatment for diabetic cardiomyopathy
Source: Front Cardiovasc Med. 2025 Mar 14;12:1454647. doi: 10.3389/fcvm.2025.1454647 (PMC11949964; doi:10.3389/fcvm.2025.1454647)
Supplement: Supplementary file 2 [file Datasheet2.docx]

TABLE Details of treatment

| Author | Components of Chinese herbal medicine | Common western medicine | Specific therapy |
| --- | --- | --- | --- |
| Yu Huichao 2019(20) | Rehmannig glutinosa Libosch. [Orobanchaceae; Rehmanniae radix praeparata] 250g，Glycyrrhiza uralensis Fisch. ex DC. [Fabaceae; Glycyrrhizae radix et rhizoma]60g，Zingiber offcinale Rosc. [Zingiberaceae; Zingiberis Rhizoma Recens]45g, Neolitsea cassia (L.) Kosterm. [Lauraceae; Cinnamomi Ramulus] 45g，Asini Corii Colla [Equus asinus L, Equidae] 30g, Ophiopogon japonicus (Thunb.) Ker Gawl. [Asparagaceae; Ophiopogonis radix]30g, Panax ginseng C.A.Mey. [Araliaceae; Ginseng radix et rhizoma] 30g，Cannabis sativa L. [Cannabaceae; Cannabis Fructus] 20g. | Controlling blood glucose, blood pressure, blood lipids, etc. | Oral administration of captopril 12.5mg, 3 times/day; Intravenous drip of vasodilators. |
| Huang Ruixia 2018(21) | Rehmannig glutinosa Libosch. [Orobanchaceae; Rehmanniae radix praeparata] 250g，Glycyrrhiza uralensis Fisch. ex DC. [Fabaceae; Glycyrrhizae radix et rhizoma]60g，Zingiber offcinale Rosc. [Zingiberaceae; Zingiberis Rhizoma Recens] 45g, Neolitsea cassia (L.) Kosterm. [Lauraceae; Cinnamomi Ramulus] 45g，Asini Corii Colla [Equus asinus L, Equidae] 30g,Ophiopogon japonicus (Thunb.) Ker Gawl. [Asparagaceae; Ophiopogonis radix] 30g, Panax ginseng C.A.Mey. [Araliaceae; Ginseng radix et rhizoma] 30g，Cannabis sativa L. [Cannabaceae; Cannabis Fructus] 20g. | Controlling blood glucose, blood pressure, blood lipids, etc. | Oral administration of captopril 12.5mg, 3 times/day; Intravenous drip of vasodilators. |
| Ma Xiaojiang 2017(22) | Rehmannig glutinosa Libosch. [Orobanchaceae; Rehmanniae radix praeparata] 250g，Glycyrrhiza uralensis Fisch. ex DC. [Fabaceae; Glycyrrhizae radix et rhizoma]60g，Zingiber offcinale Rosc. [Zingiberaceae; Zingiberis Rhizoma Recens] 45g, Neolitsea cassia (L.) Kosterm. [Lauraceae; Cinnamomi Ramulus] 45g，Asini Corii Colla [Equus asinus L, Equidae] 30g,Ophiopogon japonicus (Thunb.) Ker Gawl. [Asparagaceae; Ophiopogonis radix] 30g, Panax ginseng C.A.Mey. [Araliaceae; Ginseng radix et rhizoma] 30g，Cannabis sativa L. [Cannabaceae; Cannabis Fructus] 20g. | Controlling blood glucose, blood pressure, blood lipids, etc. | Oral administration of captopril 12.5mg, 3 times/day; Intravenous drip of vasodilators. |
| Wang Cuixia 2015(23) | Glycyrrhiza uralensis Fisch. ex DC. [Fabaceae; Glycyrrhizae radix et rhizoma]60 g，Zingiber offcinale Rosc. [Zingiberaceae; Zingiberis Rhizoma Recens] 45 g，Panax ginseng C.A.Mey. [Araliaceae; Ginseng radix et rhizoma] 30 g，Rehmannig glutinosa Libosch. [Orobanchaceae; Rehmanniae radix praeparata] 250 g，Neolitsea cassia (L.) Kosterm. [Lauraceae; Cinnamomi Ramulus] 45 g，Asini Corii Colla [Equus asinus L, Equidae] 30 g，Ophiopogon japonicus (Thunb.) Ker Gawl. [Asparagaceae; Ophiopogonis radix] 30 g，Cannabis sativa L. [Cannabaceae; Cannabis Fructus] 20 g. | Administration of nitroglycerin 5 mg and dopamine 20mg; Intravenous drip of insulin 4U+5% glucose 250ml; Intravenous drip of furosemide 20~40 mg. | NA |
| Li Songyan 2014(24) | Not specified | Controlling blood glucose, blood pressure, blood lipids, etc. | Oral administration of captopril 12.5mg, 3 times/day; Intravenous drip of vasodilators. |
| Wang Cuixia 2013(25) | Glycyrrhiza uralensis Fisch. ex DC. [Fabaceae; Glycyrrhizae radix et rhizoma] 45g，Zingiber offcinale Rosc. [Zingiberaceae; Zingiberis Rhizoma Recens] 45 g，Panax ginseng C.A.Mey. [Araliaceae; Ginseng radix et rhizoma] 30 g，Rehmannig glutinosa Libosch. [Orobanchaceae; Rehmanniae radix praeparata] 250 g，Neolitsea cassia (L.) Kosterm. [Lauraceae; Cinnamomi Ramulus] 45 g，Asini Corii Colla [Equus asinus L, Equidae] 30 g，Ophiopogon japonicus (Thunb.) Ker Gawl. [Asparagaceae; Ophiopogonis radix] 30 g，Cannabis sativa L. [Cannabaceae; Cannabis Fructus] 20 g. | Controlling blood glucose, blood pressure, blood lipids, etc. | Oral administration of captopril 12.5mg, 3 times/day; Intravenous drip of vasodilators. |
| Du Liwei 2016(26) | Glycyrrhiza uralensis Fisch. ex DC. [Fabaceae; Glycyrrhizae radix et rhizoma] 60 g，Rehmannig glutinosa Libosch. [Orobanchaceae; Rehmanniae radix praeparata] 250 g，Zingiber offcinale Rosc. [Zingiberaceae; Zingiberis Rhizoma Recens] 45 g，Ophiopogon japonicus (Thunb.) Ker Gawl. [Asparagaceae; Ophiopogonis radix] 30 g， Asini Corii Colla [Equus asinus L, Equidae] 30 g，Neolitsea cassia (L.) Kosterm. [Lauraceae; Cinnamomi Ramulus] 45 g，Panax ginseng C.A.Mey. [Araliaceae; Ginseng radix et rhizoma] 30 g，Cannabis sativa L. [Cannabaceae; Cannabis Fructus] 20 g. | Controlling blood glucose, blood pressure, blood lipids, etc. | Oral administration of captopril 12.5mg, 3 times/day; Intravenous drip of vasodilators. |
| Li Dongshan 2013(27) | Cannabis sativa L. [Cannabaceae; Cannabis Fructus] 20g, Ophiopogon japonicus (Thunb.) Ker Gawl. [Asparagaceae; Ophiopogonis radix] 30g, Asini Corii Colla [Equus asinus L, Equidae] 30g, Neolitsea cassia (L.) Kosterm. [Lauraceae; Cinnamomi Ramulus] 45g, Rehmannig glutinosa Libosch. [Orobanchaceae; Rehmanniae radix praeparata] 250 g, Panax ginseng C.A.Mey. [Araliaceae; Ginseng radix et rhizoma] 30 g, Zingiber offcinale Rosc. [Zingiberaceae; Zingiberis Rhizoma Recens] 45 g, Glycyrrhiza uralensis Fisch. ex DC. [Fabaceae; Glycyrrhizae radix et rhizoma]60 g. | Controlling blood glucose, blood pressure, blood lipids, etc. | Oral administration of captopril 12.5mg, 3 times/day; Intravenous drip of vasodilators. |
| Wu Bingyu 2019(28) | Cannabis sativa L. [Cannabaceae; Cannabis Fructus] 20g, Ophiopogon japonicus (Thunb.) Ker Gawl. [Asparagaceae; Ophiopogonis radix] 30g ,Asini Corii Colla [Equus asinus L, Equidae] 30 g,Neolitsea cassia (L.) Kosterm. [Lauraceae; Cinnamomi Ramulus] 45g,Rehmannig glutinosa Libosch. [Orobanchaceae; Rehmanniae radix praeparata] 250g,Panax ginseng C.A.Mey. [Araliaceae; Ginseng radix et rhizoma] 30g , Zingiber offcinale Rosc. [Zingiberaceae; Zingiberis Rhizoma Recens] 45g, Glycyrrhiza uralensis Fisch. ex DC. [Fabaceae; Glycyrrhizae radix et rhizoma] 60 g. | Administration of nitroglycerin 5 mg and dopamine 20mg; Intravenous drip of insulin 4U+5% glucose 250ml; Intravenous drip of furosemide 20~40 mg. | NA |
