# Supplementary material for: Integrated meta-analysis and network pharmacology analysis: evaluation of Zhigancao decoction as treatment for diabetic cardiomyopathy
Source: Front Cardiovasc Med. 2025 Mar 14;12:1454647. doi: 10.3389/fcvm.2025.1454647 (PMC11949964; doi:10.3389/fcvm.2025.1454647)
Supplement: Supplementary file 3 [file Datasheet3.docx]

EFF

LVEDV

LVESV

LVEF

LVDD

CD

SBP

DBP

AD

低血压
